# Acid Drop-Out in Carbon Capture and Transport Systems: Causes, Consequences, and Countermeasures

**DOI:** 10.3390/ma19142934

**Published:** 2026-07-08

**Authors:** Garima Mittal, Shiladitya Paul

**Affiliations:** 1 Independent Researcher, Saint Louis, MO 63112, USA; 2 Materials Innovation Centre, School of Engineering, University of Leicester, Leicester LE1 7RH, UK; 3 Materials Performance and Integrity Technology Group, TWI Ltd., Cambridge CB21 6AL, UK

**Keywords:** carbon capture and transport, acid dropout corrosion, impurity reactions, pipeline integrity

## Abstract

**Highlights:**

- Reactive impurities present in CO_2_ streams can produce liquid precipitates of H_2_SO_4_ and HNO_3_, causing severe pipeline corrosion.
- Impurity profile depends on the CO_2_ capture route, and the mixing of the oxidizing and reducing streams at the CCS hubs exaggerates the reactive phase behavior.
- Mitigation strategies, including coatings, CRAs, chemical inhibitors, and integrated thermodynamic–kinetic modeling, can help monitor acid dropout corrosion.
- Thermodynamic models, kinetic modeling, and online monitoring can help predict acid dropout, but non-ideal mixing, nucleation delays, and transient events still remain challenging.

**Abstract:**

Carbon capture and storage (CCS) technology can play an important role in meeting net-zero ambitions; however, its successful deployment depends on the transport and storage infrastructure for CO_2_, as they are the backbone of the carbon management industry. Among the key integrity threats for dense-phase and supercritical CO_2_ pipelines, acid precipitation or dropout in CO_2_-rich streams containing reactive impurities (SO_x_, NO_x_, H_2_S, H_2_O, O_2,_ etc.) is one of the most serious. These impurities can alter phase behavior, promote formation of highly acidic liquid-phase condensates, and trigger severe localized corrosion and rapid wall-thickness loss. This review focuses on understanding the effects of specific combinations of impurities on CO_2_ phase envelopes, acid formation, and corrosion mechanisms in pipelines under realistic flow and operating conditions. It further assesses mitigation and design strategies, including impurity specification and control, deep dehydration, operational envelope management, corrosion-resistant alloys, internal linings and advanced coatings, and emerging modeling tools for predicting corrosive dropout. The knowledge gap in long-term performance under multi-impurity conditions, thermo-hydraulic transients, and coupled corrosion damage is highlighted. Additionally, the importance of future experimental, modeling, and standards development work to enable safe, cost-effective material solutions for CCS technology deployment is proposed.

## 1. Introduction

Around the globe, countries are becoming proactive in implementing carbon capture technologies to reduce their carbon emissions and transition to a low-carbon economy. For instance, the Northern Lights project, owned in equal shares by TotalEnergies, Equinor, and Shell, is the first project in the world to allow industrial companies to transport CO_2_ via ships and sequester their CO_2_ emissions with a capacity of handling up to 1.5 million tons of CO_2_ per year [1]. Northern Lights has also developed a detailed liquid CO_2_ quality specification that sets tight limits on water and acid-forming impurities, such as SO_x_, NO_x_, O_2_, and H_2_S, to protect material integrity and operability.

Despite increased momentum, deployment of carbon capture and storage (CCS) technology faces various issues that limit its universal acceptance, including high infrastructure costs, low CO_2_ capture efficiency, the energy penalty during capture, and unclear and inefficient policy and regulatory frameworks. According to a recent Institute of Energy Economics and Financial Analysis (IEEFA) study, 10 out of 13 leading carbon capture projects failed or did not meet their designed capabilities [2]. For instance, the world’s only carbon capture plant associated with a coal-fired power plant, Boundary Dam in Canada, captured around 44% of Boundary Dam’s emissions in 2021, while it has the capacity to capture 90% (1 million tons) of CO_2_ annually. It happened because one of its compressor motors failed, leading to the facility shutdown for several months [3]. Likewise, the Petra Nova CCS project (started in 2017) was designed to capture ~90% (1.4 million metric tons) of CO_2_ from a 240 MW slipstream of flue gas annually. According to a report by the Institute for Energy Economics and Financial Analysis (IEEFA), the plant suffered from chronic, significant outages, failing to operate for over 367 days between its 2017 start and 2020 closure [4].

To establish CCS technology as a basis for a CO_2_ emissions reduction strategy, it is essential to invest in research and development, improve process efficiency, and implement supportive policies and regulatory frameworks. Although CO_2_ has been transported safely for decades in enhanced oil recovery (EOR) systems, these pipelines typically handle CO_2_ that is relatively pure and tightly dehydrated. Whereas, in CCS, CO_2_-rich streams (CO_2_ with impurities) from combustion and industrial sources contain a wider range of impurities (SO_x_, NO_x_, O_2_, H_2_S, solvent residues, etc.) at low concentrations. These impurities can react among themselves and trace H_2_O to form strong acids, leading to acid precipitation or dropout (hereafter, acid precipitation is referred to as acid dropout) and a much more aggressive corrosion environment for pipeline material [5]. Understanding how specific impurity combinations in CO_2_-rich mixtures affect acid formation, phase behavior, and corrosion mechanisms is critical for safe operations and cost-effective material solutions for CCS technology deployment.

In this context, this article presents a critical narrative review of acid drop-out in CO_2_-rich CCS systems, covering its possible reasons, effects on pipeline materials, and mitigation strategies, including impurity control, dehydration, coatings, and material selection. A brief overview is also provided of the CO_2_ sources, capture technologies, and current CO_2_ quality specifications.

To identify and evaluate published studies on acid dropout in carbon capture and storage, a structured literature search was conducted prior to drafting the manuscript. The scientific literature search was performed using the Scopus, Web of Science, and Google Scholar databases. The search was conducted using combinations of keywords like “CO_2_ corrosion”, “impure CO_2_”, “dense-phase CO_2_”, “acid dropout”, “pipeline corrosion”, “sulfuric acid formation”, “nitric acid formation”, “CO_2_ transport”, and “impurity interactions”. The literature search was primarily limited to journal articles published between 2010 and 2026, but older papers were included when they provide a fundamental understanding and background of the processes. Then, the literature was selected based on its relevance to impurity-dependent phase behavior or acid formation mechanisms in CO_2_ streams, thermodynamic and kinetic modeling, corrosion of pipeline materials (steels or corrosion-resistant alloys) in dense CO_2_ with impurities, and CO_2_ specification limits and corrosion-mitigation guidelines for CO_2_ pipelines. In some places, non-peer-reviewed articles were also included to present specific project-related data or recommended practices. Then, full texts were reviewed to confirm eligibility, and studies that focus on conventional CO_2_ corrosion in oil and gas production and are not related to dense-phase CO_2_ transport or impurity-related acid formation were not included in this review. This structured but non-systematic methodology helped us to obtain a comprehensive yet focused assessment of the current state of understanding of acid dropout in CCS systems.

### 1.1. Carbon Capture Processes

All carbon capture routes consist of three steps: capture (including partial drying and impurity removal), compression, and transportation. The technologies (amine absorption, physical solvents, membranes, etc.) and upstream process conditions used in these capture methods determine the types and levels of impurities captured with CO_2_, including H_2_O, SO_x_, NO_x_, O_2_, H_2_S, CO, hydrocarbons, and trace organics, causing acid formation and dropout.

This section summarizes the basic principles of carbon capture processes and the factors influencing impurities in captured CO_2_ streams.

#### 1.1.1. Post-Combustion Carbon Capture Process

Post-combustion carbon capture is a widely used method that captures CO_2_ from flue gases after combustion, as the name suggests. Despite having low CO_2_ concentration (3–20%) and low CO_2_ partial pressure (0.03–0.2 bar) [6,7], the post-combustion carbon capture method is considered a more feasible option due to its retrofit nature to the existing plants and scalability. Commonly used technologies for separating CO_2_ from flue gas in post-combustion are chemical absorption, adsorption, and membrane separation. Among these, chemical absorption using aqueous amine solutions such as monoethanolamine (MEA), which captures around 85–90% of CO_2,_ is a more technologically mature CO_2_ separation technique for power plants. Companies like Mitsubishi Heavy Industries (Tokyo, Japan), Fluor Corporation (Irving, TX, USA), HTC Purenergy Inc. (Regina, SK, Canada), Aker Carbon Capture (Lysaker, Norway), and Kerr-McGee/ABB Lummus (Houston, TX, USA) are examples of industries using this post-combustion carbon capture method [8]. Several other technological approaches, including sorbents and membranes, also have the potential to improve post-combustion capture and might outperform solvents over time; however, further R&D is required to surpass the shortcomings of each of them.

Commonly present impurities in the CO_2_ stream captured from the post-combustion process include NO_x_, SO_2_, H_2_S, trace elements, and particulate matter. Impurity levels in the CO_2_ stream depend on a variety of factors such as fuel type and its properties, including trace element level, ash properties (sulfur content in ash), and volatile matter, insertion of excessive air in the boiler, air ingress or inert components in air, temperature range of the furnace, flue gas treatment (flue gas desulfurization (FGD), SO_x_ removal efficiency, SO_2_/SO_3_ conversion), and post-combustion NO_x_ controls like selective catalytic reduction (SCR) and electrostatic precipitator (ESP) removal efficiency [9,10]. Therefore, a better understanding of the factors influencing impurities in post-combustion carbon capture processes is essential for optimizing the performance of the post-combustion carbon capture system and for defining downstream CO_2_ stream quality specifications [9].

#### 1.1.2. Pre-Combustion Carbon Capture Process

In this method, CO_2_ is captured before combustion by converting fuel feedstocks (coal or natural gas) into synthesis gas (syngas; a mixture of CO, H_2_, CO_2,_ and H_2_O) via gasification, steam reforming, auto-thermal reforming, or partial oxidation. It is mainly employed in integrated gasification combined cycle (IGCC) industrial processes. This process involves the water–gas shift (WGS) reaction, which converts CO into CO_2_ and produces more hydrogen from water, followed by the removal of CO_2_ from the hydrogen-rich syngas via physical or chemical absorption before combustion. Since the CO_2_ concentration in the gaseous mixture is relatively high (~15–60% (dry basis) at a total pressure of 2–7 MPa), physical solvents like mixtures of dimethyl ethers of polyethylene glycols (often marketed as Selexol) and low-temperature methanol-based systems (like Rectisol) are preferred over chemical solvents for carbon capture [11,12]. Once CO_2_ is captured, the hydrogen-rich fuel gas is used directly for power and heat generation, such as in gas turbines. Even though the pre-combustion process can reduce carbon capture costs by 38–45% and 21–24% compared to the post-combustion and oxy-fuel combustion processes, respectively, the base capital cost required to upgrade existing facilities complicates its commercialization [13]. One great example of this is Mississippi Power’s ‘Kemper Project,’ which was supposed to gasify lignite coal and capture CO_2_ emissions from the syngas pre-combustion. But because of project delays and increased costs, it was ultimately abandoned [14]. On the other hand, Dakota Gas’s Great Plains Synfuels Plant (North Dakota, USA) gasifies approximately 18,000 tons of lignite, generating 170 million cubic feet of syngas and capturing up to 3 million tons of CO_2_ per year. The captured CO_2_ is compressed and transported to Saskatchewan, Canada, through pipelines, where it is utilized in enhanced oil recovery operations [15].

Impurities in the pre-combustion process-generated CO_2_ stream are typically minimal and are mainly affected by the steps involved in generating and processing the syngas into an H_2_-rich stream. The purity level of the supplied oxygen affects the N_2_ and Ar levels in the CO_2_ product stream. In the reducing atmosphere of the gasifier, most fuel sulfur is converted to gas-phase sulfur species such as H_2_S (with smaller amounts of COS), which can be carried forward to the CO_2_-rich product stream if not removed in the acid–gas treatment step. Also, gasifier temperature, pressure, equivalence ratio, and water or steam input control the syngas properties as well as CO_2_ purity [9].

#### 1.1.3. Oxy-Fuel Combustion Carbon Capture Process

Oxy-fuel combustion is a variation in the post-combustion carbon capture process that involves burning fuel in pure oxygen rather than air, resulting in a much higher CO_2_ concentration (yield > 95%). To limit the maximum temperature during the process, a fraction of the flue gases is reinjected into the combustion process. Compared with post-combustion, oxy-fuel combustion requires three main additional units: a cryogenic air separation unit (ASU) for high-purity oxygen, a CO_2_ compression and purification unit for removing water, particulate matter, and other pollutant gases, and a flue gas recycle system. Generally, a cryogenic ASU is required to supply pure oxygen (90–95% *v*/*v*) on a large scale, which makes the process expensive and slows its implementation in coal-based power plants. However, oxy-fuel combustion is considered a highly energy-efficient and promising carbon capture process because CO_2_ in the flue gas, which is mostly CO_2_ and H_2_O, can be easily captured by cooling and compressing. Compared to the post-combustion process, the oxy-fuel process can significantly reduce the energy penalty and the capture system size because the flue gas is CO_2_-rich, and, hence, lowers solvent regeneration and compression loads, especially at high capture rates [16]. Many techno-economic studies indicate that oxy-fuel set-ups can be competitive with, or in some cases cheaper than, post-combustion capture for new builds and selected retrofit options, depending on the oxygen-supply technology and site-specific constraints [17,18].

A well-known pilot example is Vattenfall’s 30 MW_th_ Schwarze Pumpe. Some examples of this process are oxy-fuel CO_2_ purification and a sequestration plant in Schwarze Pumpe, Germany, which demonstrated the full oxy-fuel combustion, CO_2_ compression, and purification chain, and supplied ~1500 tons of captured CO_2_ for injection at the Ketzin storage site [19]. Vattenfall discontinued its CCS research program in 2014 due to cost and energy penalty concerns. More recently, NET Power’s 50MWth Allam-cycle demonstration plant in La Porte, Texas, has shown an oxy-fuel, CO_2_-working-fluid power cycle with net-zero atmospheric emissions and is being scaled to the first utility-scale plant integrated with CO_2_ sequestration [20,21].

Impurity levels in the CO_2_ stream captured from the oxy-fuel plant vary with power plant configuration, coal combustion, mode of operation, and the used CO_2_ compression and purification unit, and these impurities can be introduced as combustion byproducts, excess oxygen, air ingress, or inert components in air from ASU [9]. For instance, during flue gas recycling, flue gas desulfurization (FGD) is introduced, which helps reduce NOx and SO_2_ concentrations in emissions. Similarly, the purity of oxygen obtained through ASU generally lies between 90 and 95% *v*/*v*, which influences the N_2_ and Ar levels in the raw CO_2_ gas stream. The use of purer oxygen (~99% *v*/*v*) further increases operating costs. In addition, impurity content and level in the CO_2_ stream are affected by coal properties, including sulfur content, ash properties, and trace element levels. Coal with a high sulfur content is responsible for high SO_x_ levels in the CO_2_ stream; however, these levels can be controlled by varied oxyfuel combustion configurations and arrangements for the FGD unit. For instance, for flue gas generated from coal with up to 0.5% sulfur, treatment with FGD outside the recycle loop and the flue gas cooler is sufficient. If the sulfur content is 0.5–1.0%, i.e., an intermediate level, an arrangement in which FGD is located inside the recycle loop is useful, removing SO_2_ before the flue gas returns to the boiler. For high-sulfur-content (more than 2%) coal, a high-efficiency wet FGD inside the recycle loop with either an additional spray drying absorption system prior to fabric filter particulate removal or a wet ESP after the flue gas cooler should be considered [22]. Similarly, ash properties affect the particulate content in the CO_2_ stream, except when ash has a high level of calcium, which can act as a sorbent for sulfur, reducing gas-phase SO_x_. Excess oxygen in the boiler and air leakage into the boiler and other parts will influence the N_2_, O_2_, and Ar concentrations in the CO_2_ stream. Furnace temperature also affects NO_x_ formation and post-combustion NO_x_ controls like selective catalytic reduction (SCR) can control NO_x_ levels in the CO_2_ stream; however, the use of ammonia in the process might introduce further impurities into the CO_2_ product stream [9].

#### 1.1.4. Direct Air Carbon Capture

Direct air capture (DAC), or direct CO_2_ capture, unlike others, involves separating CO_2_ directly from the atmosphere rather than targeting CO_2_ emissions from specific point sources or locations. Direct carbon capture can be categorized as solid-sorbent- or liquid-solvent-based. Although capturing CO_2_ using DAC is more expensive and energy-intensive as CO_2_ in the atmosphere is much more diluted (~400 ppm(v) in air) than from point sources, the captured CO_2_ via DAC does not need expensive purification as its impurities are primarily N_2_, O_2_, and water vapor from the atmosphere, and their levels vary with air quality, humidity, temperature, and the performance of capture materials or technologies.

According to IEA’s report, nine direct carbon capture facilities are in progress (Table 1), with an estimated capacity to deploy almost 3 MtCO_2_ by 2030, which is >380 times the current capture rate [23].

### 1.2. Impurities in CO_2_ Streams

Numerous anthropogenic and natural sources contribute to CO_2_ emissions, and depending on the carbon capture method and emission source, the captured CO_2_ stream exhibits a distinct composition of impurities. Table 2 summarizes the typical impurity profiles across various CO_2_ capture routes and the factors affecting impurity composition.

Beyond these qualitative profiles, published studies provide different acid-forming impurity and non-condensable impurity ranges in CO_2_ product streams from different capture routes. The purity order of CO_2_ captured from main carbon capture technologies are oxy-fuel (double flashing; ~96%) < pre-combustion (~98%) < post-combustion (~99.6%) [9]. For post-combustion capture, the main impurities are N_2_, H_2_O and O_2_. After FGD/SCR and solvent regeneration, a post-combustion CO_2_ product stream can reach more than 99% pure CO_2_ with −0.1–4 vol% range of N_2_, O_2_, and Ar together. Water is controlled through dehydration (100–640 ppm(v)), and SO_2_ and NO_x_ often range from <10 ppm to a few hundred ppm, depending on the used fuel and cleanup efficiency. The post-combustion stream, if not strictly desulfurized, and dehydrated, can pose a relatively high acidification risk.

Captured CO_2_ from pre-combustion capture is usually 95–99% pure, and usually free from O_2_. The main impurity concern from pre-combustion is residual H_2_S/COS (up to 34,000 ppm(v)), along with H_2_ (20–30,000 ppm(v)), CO (up to 2000 ppm(v)), and CH_4_ (up to 112 ppm(v)). Since these streams are strongly reducing and contain little or no NO_2_/O_2_, the possibility of NO_2_-driven H_2_SO_4_/HNO_3_ formation is low. When pre-combustion CO_2_ is mixed with more oxidizing streams in CCS hubs, the acidification risk becomes high. Captured CO_2_ from oxy-fuel systems, after compression and purification, can reach more than 95–99 vol% purity, and non-condensable impurities (N_2_, O_2_, and Ar) can be present in the 0.1–3 vol% range. The residual SO_2_ and NO_x_ levels can vary from <10 ppm to several hundred ppm depending on sulfur content in coal, ASU purity and FGD/SCR configuration. If improperly cleaned, the risk of acidification increases. CO_2_ captured through DAC typically contains mainly N_2_, O_2_ and water, and has very low levels of sulfur- and nitrogen-containing species. The impurity levels vary with air quality, humidity and sorbent/solvent properties. Therefore, the acidification risk in DAC-captured CO_2_ is much lower, unless it later mixed with oxidizing streams in CCS hubs.

Overall, if post-combustion and oxy-fuel combustion CO_2_ streams are not properly cleaned, they can reach up to or above acidification thresholds, while pre-combustion and DAC CO_2_ streams are usually below acidification thresholds unless they are mixed with other sources in CCS hubs.

Along with capture routes, it is essential to understand CO_2_ sources and their associated impurities to design efficient carbon capture systems.

#### 1.2.1. Fuel- and Capture-Related Impurities

Depending on their origin, impurities can be categorized as those generated by fuel oxidation, excess oxidant/air ingress, and process fluids. Typically, the coal and biomass combustion process generates more impurities than natural gas combustions, which includes H_2_O, SO_x_, NO_x_ and halogens (from complete oxidation), CO, H_2_S, COS, NH_3_, HCN (from partial oxidation due to fuel-rich situations), hydrocarbons and hydrogen (from fuel devolatilization with heating), chlorides, sulfates and potassium and sodium hydroxides (from biomass fuel), trace metals (contained in fuel), ash and soot with polycyclic aromatic hydrocarbon (PAH) precursors particulates, and O_2_, N_2_, and Ar (from excess oxidant/air ingression) [9]. Sometimes, process fluids such as monoethanolamine (MEA), glycols, and solvents based on dimethyl ethers of polyethylene glycol can also introduce solvent-specific degradation products or entrained solvent into the CO_2_ stream during pre- or post-combustion capture.

With the fuel type and combustion conditions, the composition of flue gas emissions and the presence of impurities in the captured CO_2_ also vary. Flue gas derived from coal-fired power plants usually contains impurities including CO_2_, N_2_, O_2_, H_2_O, SO_2_, NO_x_, particulate matter, and trace elements, and the amount of these impurities varies with fuel composition. In case of natural gas combustion, flue gas stream derived from natural gas combined cycle (NGCC) power plant contains lower levels of impurities, including around N_2_ (~74.4%), O_2_ (~12.4%), H_2_O (~8.4%), CO_2_ (~3.9%) and Ar (~0.9%), exhibiting the higher air-to-fuel ratio and lower carbon and sulfur content of natural gas [24]. However, a significantly lower CO_2_ partial pressure in NGCC flue gas (~40 mbar) than that of coal flue gas (~150 mbar) makes post-combustion CO_2_ capture from NGCC technically more challenging.

#### 1.2.2. Biomass Co-Firing and Ash-Related Impurities

Biomass, an alternative to fossil fuels (sometimes co-fired with them), combined with carbon capture technology, can contribute to net negative emissions, as CO_2_ stored in biomass through photosynthesis is captured rather than emitted into the environment. Since biomass contains less sulfur, fixed carbon, and fuel-bound nitrogen, but more oxygen, specifications of the capture CO_2_ stream vary with the fuel chemical composition, depending if the biomass is used as a whole or in combination with coal [25]. Many studies suggest that using a combination of coal and biomass as a fuel emits fewer pollutants than a coal-powered plant. For instance, Kommalapati et al. found that CO_2_, CO, SO_2_, PM2.5, NO_x_, and VOC emissions could be reduced by 13.5%, 6.4%, 9.5%, 9.2%, 11.6%, and 7.7%, respectively, when 15% of coal is replaced with forest residue [26]. Apart from this, co-firing biomass fuels with coal can help reduce the oxygen content in the flue gas by up to two times compared with pure coal combustion [27]. Despite this, the problem is the abundance of alkaline minerals, including K, Na, Mg, and Ca, in biomass, which can cause corrosion, slagging, and fouling in plant components by condensing on fly ash and forming a sticky layer. Depending on fuel composition and environmental conditions, the chemical forms of the released alkali metals vary. For instance, the presence of Cl and S in the fuel can facilitate the formation of alkali chlorides and alkali sulfates during combustion. Similarly, at different reaction temperatures and pressures, alkali transformation paths and rates vary [28]. Fly ashes generated from biomass combustion comprise different compounds than ashes derived from burning coal, as the first one has a lower amount of aluminum, silicon, iron, and sulfur, and an increased content of calcium, chlorine, potassium, magnesium, sodium, oxygen, and phosphorus [29,30]. These ash particles and alkali metals may threaten the integrity of carbon capture infrastructure; hence, further R&D efforts are required to address impurity-related challenges.

The application of a carbon capture unit to any industrial unit depends on technical feasibility, which is determined by the plant’s layout. Table 3 summarizes the typical CO_2_ concentrations in flue gases from various sources.

#### 1.2.3. Industrial Source-Related Impurities

Some industrial processes, including ammonia production, natural gas processing, and synthetic fuel production, already have a CO_2_ removal or capture step as an integral part of the process, emitting relatively unpolluted CO_2_. The main industrial sources of CO_2_ emissions are discussed in the following section, but since the data related to the range and levels of impurities in CO_2_ capture from various industrial processes is not fully available, the composition of the emitted CO_2_ stream is discussed where possible.

##### Iron and Steel Production

This industry is the major source of CO_2_ emissions and an energy-intensive sector that accounts for 2.6 Gt of CO_2_ (7% of the global anthropogenic CO_2_ emissions) annually [35]. The majority of CO_2_ emissions originating from steel production come from blast furnace stacks, requiring >70% of the total operation energy, followed by the coke plant. The CO_2_ emissions arising from the blast furnace and basic oxygen furnace (BF-BOF) process are higher (2.2 tons of CO_2_ emissions per ton of crude steel) than the direct reduced iron and electric arc furnace (DRI-EAF) process (1.4 tons of CO_2_ emissions per ton of crude steel), ascribed to the use of electricity in the latter [36]. Typically, blast furnace gas is composed of N_2_ (55.19%), CO_2_ (21.27%), CO (20.78%), and H_2_ (2.76%) [37]. Other than blast furnaces, sintering, coke ovens, pellets, and other furnaces also emit CO_2_ along with other pollutants, including SO_x_, NO_x_, H_2_S, HCN, NH_3_, particulate matter, heavy metals, etc. [38]. When the carbon capture process is used on this flue gas, the captured CO_2_ is likely to contain similar impurities.

##### Construction Industry

There is a huge contribution to greenhouse gas emissions in the cement industry, attributed to the calcination process (~50%), fuel combustion for energy (~40%), and the remaining 10% from manufacturing operations [39]. However, since 2018, CO_2_ emissions from the cement industry have remained relatively constant, at just under 0.6 t CO_2_/tons of cement produced [40]. Primarily, NO_x_, SO_2_, CO, and CO_2_ are emitted from cement production, but a small volume of VOCs, NH_3_, chlorine, and HCl may also be observed. The CO_2_ concentration in flue gases from the cement industry is around 15–30% by volume.

##### Hydrogen and Ammonia Production

Globally, the Haber–Bosch process is the main industrial process for producing ammonia, involving the reaction between nitrogen from air and hydrogen derived from natural gas, coal, or other hydrocarbons. Typically, hydrogen is produced through steam reforming, auto-thermal reforming, partial oxidation, and gasification, depending on the economics, feedstock source, and plant flexibility, resulting in CO_2_ emissions as a byproduct. Globally, 2.4 tons of CO_2_ per ton of ammonia production is generated, which is nearly 2×of the CO_2_ emissions arising from crude steel production and 4× that of cement, on a direct CO_2_ emission basis [41]. The above-mentioned methods of hydrogen production include the use of solid-fuel gasification or natural gas reforming technologies, producing a syngas that is purified by a gas cleanup step to generate a reformed syngas mix of H_2_. The water–gas shift reaction converts syngas to a mixture of CO_2_ and hydrogen, followed by CO_2_ removal, producing a purified stream of H_2_. Capturing 80–85% of CO_2_ in hydrogen production using natural gas through the steam–methane reformation method adds around 25–30% more to the production cost [42,43].

##### Natural Gas Processing

To fulfill the pipeline specifications and environmental regulations, raw natural gas, which contains methane, ethane, propane, butanes, and heavier hydrocarbons along with other constituents, including CO_2_, H_2_S, H_2_O, N_2_, and trace components, undergoes processing [44]. During processing, H_2_S is removed, the dew-point of the gas stream is adjusted to remove water and hydrocarbons from the stream to avoid liquid dropout in pipelines, and CO_2_ is separated. To capture CO_2_ from natural gas containing high CO_2_ content, various technologies can be employed, including pressure swing adsorption (PSA), cryogenic distillation, membrane permeation, physical and chemical absorption, and gas–liquid membrane contactor [45]. Captured CO_2_ is often compressed and transported using pipelines to injection sites for geological storage or industrial plants for utilization in enhanced oil recovery (EOR) or other applications. The world’s first and longest-running commercial CO_2_ storage project is Sleipner, which covers capturing CO_2_ from natural gas production and injecting it into a deep saline aquifer for permanent geological storage. By 2016, this project had injected approximately 16 million tons of CO_2_ into the Utsira sandstone formation since 1996 [46]. Long-term monitoring has demonstrated stable plume evolution and secure storage over more than two decades of operations [47].

##### Lime Production

Lime production is done through the calcination of limestone, dolomite, or other mineral materials at temperatures between 900 °C and 1200 °C, occurring in vertical and rotary kilns fired by gas, oil, coal, coke, or some types of secondary fuels (e.g., oil, plastics, paper). Commonly released pollutants from lime kilns are CO_2_, SO_x_, NO_x_, CO, volatile organic compounds, and particulate matter, depending on the used fuel’s properties, the used mineral feed’s properties, the kiln type, pollution control equipment, and the quality of the produced lime [48]. Apart from this, toxic species, including nickel, arsenic, chromium, cadmium, and HCl, are also found in emitted gases from lime kilns [49].

##### Mixed Industrial Sources and CCS Hubs

Carbon dioxide emissions originating from multiple processes and sources are challenging for carbon capture, transport, and storage technologies due to the associated range of impurities. These impurities can be distinguished based on different carbon capture methods and industrial sources. Recently, a hub approach has emerged. The hub approach proposes to capture CO_2_ emissions from multiple industrial sources; hence, each CO_2_ stream arising from different sources with a different range of CO_2_ impurities will be merged in a common pipeline or storage project that will give rise to a complex impurity profile in the mixed CO_2_ stream. Depending on the origin and processing, the CO_2_ stream can be categorized as a single-source stream or a multiple-source stream. For a single, well-defined source, the effects of impurities such as H_2_O, SO_2_, NO_x,_ and O_2_ on corrosion and operational performance are usually known, and specifications can be set accordingly; however, if not properly controlled, the reactive impurities can lead to acid formation and corrosion. Whereas a mixed source stream, arising from multiple industries, contains impurities related to gas sources such as H_2_O, NO_x_, SO_x_, O_2_, CO, and H_2_, capturing and cleaning process-specific impurities such as amine and glycols, and project-specific impurities like NH_3_, methanol, and glycol. These mixed streams with different reactive natures can induce reactions between their oxidizing and reducing components. For instance, a stream containing high levels of H_2_S-like impurities can be reducing in nature, or with O_2-_ or NO_2_-like impurities, it can be oxidizing in nature. To understand the potential risk of the different possible reactions and to avoid undesired reactions among impurities, a good understanding of CO_2_ sources is crucial when designing CCS hub infrastructure.

### 1.3. CO_2_ Specification and Impurities in Carbon Capture and Transport Systems

The effectiveness of carbon capture projects depends heavily on the quality of the captured CO_2_, which can be characterized by purity, composition, and impurity levels. The quality requirements for CO_2_ vary with its intended use, depending on compatibility with storage environments, transportation infrastructure, and utilization methods. For instance, enhanced oil recovery (EOR), chemical synthesis, and the food and beverage industries require high-purity CO_2_ to prevent mineralization, corrosion, or unwanted reactions and ensure process efficiency and product quality [50,51].

Adhering to CO_2_ specifications helps optimize the potential of CCS projects and reduce environmental pollution risks. For each project, especially when more than one carbon source is used to capture CO_2_, CO_2_ specifications need to be determined, defining an upper limit of impurity tolerance based on the infrastructure. The South West Hub project in Western Australia is an example of a CO_2_ hub, collecting CO_2_ from diverse sources in the Kwinana and Collie industrial zones for storage in the Lesueur formation in the Southern Perth Basin [52]. Usually, one CO_2_ specification is established per project for all CO_2_ suppliers, and each supplier is required to provide information on the project controls and evidence that the CO_2_ stream being distributed meets the project’s specifications. A comparison of CO_2_ specifications across major CCS projects (Table 4) reveals significant variability in impurity limits, particularly for non-condensable gases and reactive species. Some projects, such as the Northern Lights in Norway, adopt highly conservative limits, while others allow greater flexibility (e.g., Aramis, Porthos). It should be noted that these specifications are defined for transport systems and do not guarantee the absence of acid dropout or corrosion risk, as acid dropout is a system-dependent phenomenon influenced by operating conditions, transients, phase state, steel grade, etc. A meaningful benchmark of acidification risk should combine such specifications with thermodynamic/speciation calculations and lab test results obtained from controlled dense-phase CO_2_ with a defined impurity mixture. The experimental benchmark for such equivalent conditions is summarized in Section 2.1.2, where pressure, temperature, CO_2_ phase, impurity concentrations (H_2_O, O_2_, SO_2_, NO_2_, and H_2_S), corrosion rate, and observed damage are compared. This difference highlights the need for context-specific, risk-based impurity management strategies rather than overly conservative, one-size-fits-all specifications [53].

After carbon capture, the most expensive process is transporting compressed CO_2_ (~25% of the total cost of the CCS project) from the processing plant to the storage or utilization site [54]. Usually, modular (tanks carried by truck, train, or ship) or pipeline transport is used for transportation, depending on the project scale, distance, volume, infrastructure availability, cost considerations, and regulatory requirements. Pipelines are a common and efficient mode of CO_2_ transportation, offering numerous advantages, including long-distance and large-volume CO_2_ transport. Currently, in the USA, approximately 70 million tons of CO_2_ are transported each year through around 5000 miles of dedicated CO_2_ pipelines from 50 operational pipelines [55].

To ensure compatibility with transportation infrastructure, including pipelines, tankers, and compression facilities, a CO_2_-quality specification is a prerequisite. Impurities that are commonly present in CO_2_ streams include water (H_2_O), hydrogen sulfide (H_2_S), sulfur oxides (SO_x_), nitrogen oxides (NO_x_), nitrogen (N_2_), and oxygen (O_2_), which can be responsible for equipment damage, corrosion, and scaling in pipelines, and compromised transportation if present above tolerance limits. There is no detailed specification table related to failed CCS projects available in the public domain; however, from demonstration studies, it was found that when multiple sources are blended together without impurity specifications, SO_2_/NO_2_/H_2_S/H_2_O/O_2_ in the 10–100 ppm range can reach the acid dropout threshold, despite individual impurities being within their own limits. Therefore, the purity specifications for hub projects are much stricter than those for single-stream CCS projects. The latest published purity specification for CCS hub projects is H_2_O (20 ppm), H_2_S 1.3–1.5 ppm, SO_x_ 2.5–3 ppm, NO_x_ 2.5 ppm, and O_2_ 10 ppm−40 ppm [56]. Although CO_2_ itself is not explosive, a burst in a high-pressure CO_2_ pipeline can be catastrophic because the rapid release of dense, cold CO_2_ can pose a health hazard to nearby humans and animals. There was an accident near Satartia, Mississippi, in February 2020, where a CO_2_ pipeline from Denbury Enterprises burst and released CO_2_ gas, causing 49 people to be hospitalized and around 300 people to be evacuated from their homes. The reason behind this catastrophe was challenging surroundings, heavy rain for around two months, which caused pipe weld failure [57]. This incident has reinforced the need to manage both the mechanical integrity and the chemical stability of CO_2_ streams carefully as the CO_2_ network expands.

In the following section, we focus on one of the critical aspects of chemical instability, i.e., acid dropout in impure CO_2_ streams, which links the presence of trace impurities such as H_2_O, SO_x_, NO_x_, and O_2_ to localized corrosion risks in capture plants and transport infrastructure.

**Table 4:** Examples of CO_2_ quality specifications for *transport* from different projects [58,59,60,61,62].

Components	Northern Lights	Aramis		Porthos	CarbonNet	GRTgaz
	(CO_2_ Cargo, Liquefied)	Ship	Pipeline Infrastructure		Range (Low–High)	
CO_2_	Balance (Min. 99.81 mol%	Balance	>95 mol%	≥95 mol%	Balance of stream (>93.5–100 vol%)	>95 mol%
H_2_O	≤30 ppm(mol)	<30 ppm(mol)	<70 ppm(mol)	≤70 ppm(mol)	100 ppm(v)	<40 ppm(mol)
O_2_	≤10 ppm(mol)	<10 ppm(mol)	<40 ppm(mol)	≤40 ppm(mol)	2–5 vol%	<40 ppm(mol)
N_2_	≤50 ppm(mol)	-	<2.4 mol%	≤2.4 mol%		<2 mol%
H_2_	≤50 ppm(mol)	<500 ppm(mol)	<7500 ppm(mol)	≤0.75 mol%		<0.75 mol%
Ar	≤100 ppm(mol)	-	<0.4 mol%	≤0.4 mol%		<0.4 mol%
CH_4_	≤100 ppm(mol)	-	<1 mol%	≤1 mol%		<1 mol%
CO	≤100 ppm(mol)	>1200 ppm(mol)	<750 ppm(mol)	≤750 ppm(mol)	900–5000 ppm(v)	<750 ppm(mol)
O_2_ + N_2_ + H_2_ + Ar + CH_4_ + CO	-	Sum < 2000 ppm(mol)	Sum < 40,000 ppm(mol)	≤4%	-	<4 mol%
NO_x_	≤1.5 ppm(mol)	Sum < 1.5 ppm(mol)	<2.5 ppm(mol)	≤5 ppm(mol)	250–2500 ppm(v)	<10 ppm(mol)
SO_x_	≤10 ppm(mol)	Sum < 10 ppm(mol)	-	-	200–2000 (SO_2_; ppm(v))	<10 ppm(mol) (SO_3_: <0.1 ppm(mol))
H_2_S	≤1 ppm(mol)	<5 ppm(mol)	<5 ppm(mol)	-	100 ppm(v)	<9 ppm(mol)
COS	-	-	-	-	-	-
DMS	-	-	-	-	-	-
H_2_S + COS + SO_x_ + DMS	-	-	Sum < 20 ppm(mol)	-	-	-
Amine	≤10 ppm(mol)	<10 ppm(mol)	<1 ppm(mol)	-	-	-
NH_3_	≤10 ppm(mol)	<10 ppm(mol)	<3 ppm(mol)	-	-	-
Total amine compounds		-	-	≤1 ppm(mol)	-	-
CH_2_O	≤20 ppm(mol)	<20 ppm(mol)	-	-	-	-
CH_3_CHO	≤20 ppm(mol)	<20 ppm(mol)	-	-	-	-
Total aldehyde compounds	-		-	≤10 ppm(mol)	-	-
Carboxylic acids and amides	-	-	Sum < 1 ppm(mol)	≤1 ppm(mol)	-	-
Phosphorus-containing compounds	-	-	Sum < 1 ppm(mol)	≤1 ppm(mol)	-	-
C_2_H_4_	≤50 ppm(mol)	-	-	-	-	-
C_2_H_6_	≤75 ppm(mol)	-	-	-	-	-
HCN	≤100 ppm(mol)	-	<10 ppm(mol)	-	Subject to materiality threshold	-
Total VOCs (excl. MeOH, EtOH, aldehydes)	≤10 ppm(mol)	Sum < 10 ppm(mol)	Sum < 10 ppm(mol)	≤10 ppm(mol)	-	-
CH_3_OH	≤30 ppm(mol)	<40 ppm(mol)	<620 ppm(mol)	≤620 ppm(mol)	-	-
C_2_H_5_OH	≤1 ppm(mol)	<20 ppm(mol)	<20 ppm(mol)	≤20 ppm(mol)	-	-
MEG	≤0.2 ppm(mol)	-	-	-	-	-
TEG	≤0.2 ppm(mol)	-	Follow dew-point specification	-	-	-
Total glycol compounds	-	-	-	Follow dew-point specification	-	-
Aliphatic Hydrocarbons (C_3_+)	≤1100 ppm(mol)	-	<1200 ppm(mol)	≤1200 ppm(mol)	-	<1200 ppm(mol)
Aromatic Hydrocarbons (incl. BTEX)	-	-	Sum < 0.1 ppm(mol)	≤0.1 ppm(mol)	-	<0.1 ppm(mol)
Benzene, Toluene, Ethylbenzene and Xylene (BTEX)	≤0.5 ppm(mol)	-	-	-	-	-
Hg	≤0.0003 ppm(mol)	<30 ppm(mol)	-	-	-	-
Cd + Tl	-	<30 ppm(mol)	-	-	-	-
Dew-point (any liquid phase)	-	-	Sum < −10 °C (@ 20 bar)	Sum < −10 °C (@ 20 bar)	-	<−10 °C on the whole operating pressure range)
Solids, particles and/or dust	≤1.0 µm	≤1.0 µm	≤1.0 µm	-	-	-

## 2. Acid Dropout in Carbon Capture, Transport, and Storage Systems

### 2.1. Significance and Definition of Acid Dropout

#### 2.1.1. Phenomenon and Definition

Impurities in captured CO_2_ stream, even at trace levels, can significantly modify physical and thermodynamic behavior, including critical point, phase envelope, and hydrate stability region (a pressure–temperature region where CO_2_-H_2_O clathrate hydrates can form), therefore affecting the design, operation, and long-term integrity of pipeline and injection systems used in carbon capture and storage (CCS) systems [63,64,65]. CO_2_ impurities are broadly categorized as non-condensable (e.g., N_2_, O_2_, Ar, CH_4_, H_2_) and condensable (e.g., SO_2_, NO_2_, H_2_S, H_2_O, organic volatiles). Non-condensable impurities primarily alter bulk properties such as compressibility and temperature profiles, whereas condensable species can form liquid phases under pipeline operating conditions [64,66,67]. Certain impurities, such as glycol-based solvents or alcohols, may shift the phase envelope and promote water-rich phase separation, leading to water dropout even at low concentrations [68].

In this review, acid dropout refers specifically to the formation of corrosive acidic phases when species such as SO_X_, NO_X_, O_2_, H_2_S, and H_2_O react within the transported CO_2_ stream, and these formed acids exceed their solubility in the CO_2_-rich phase, leading to phase separation. This process is typically, but not always, triggered during cooling, compression/decompression, transient operation, or when the fluid composition crosses the dew-point boundary, resulting in either the condensation of water or the separate acid-rich liquid-phase formation, which can further uptake additional water from the CO_2_-rich phase and become highly corrosive. These acidic phases commonly appear as thin-wall films, microdroplets, or stratified layers, all of which pose severe risks to CCS infrastructure [69,70].

#### 2.1.2. Experimental Evidence for Acid Condensates and Corrosion

Experimental studies demonstrate that even ppm-level impurities can generate highly acidic condensates [71,72]. Such condensates have been shown to induce localized pitting, rapid general corrosion, and the dissolution of protective oxide scales, particularly in carbon steels commonly used for CO_2_ pipelines [73,74,75].

Different impurities influence corrosion behavior through distinct mechanisms. For instance, dissolved H_2_S accelerates localized corrosion by forming a relatively conductive FeS film that is cathodic to underlying steel, hence providing a cathodic surface and causing anodic dissolution at film defects and pores. While SO_2_ in the presence of water forms H_2_SO_3_, promoting Fe dissolution and rapid thinning of steel surfaces [76,77,78]. O_2_ acts as a powerful oxidizing species, enhancing corrosion via the cathodic oxygen reduction reaction (O_2_ + 2H_2_O + 4e^−^ → 4OH^−^), while NO_2_ promotes both nitration reactions and the synergistic formation of sulfuric and nitric acids through radical-driven pathways [79,80]. These impurities not only modify corrosion kinetics but also alter the structure, composition, and stability of corrosion product layers, leading to unstable or porous films that aggravate metal attack [54].

Work by Hoa et al. provides clear evidence of the severity of acid dropout in dense-phase CO_2_ systems [72]. Their experiments with steel grade L360NB exposed to mixtures of H_2_O, O_2_, NO_2_, and SO_2_ showed the formation of both H_2_SO_4_ and HNO_3_ condensates with pH values below two, leading to corrosion rates exceeding 2 mm/year under certain conditions. After diluting the actual condensate two, four, six, and ten times, the pH of the solution after saturating with CO_2_ varied from 1.93 to 2.11, 2.31, 2.46, and 2.7, respectively. The results showed that HNO_3_-dominated condensates induced faster, more uniform corrosion, whereas mixtures rich in H_2_SO_4_ or those at high HNO_3_/H_2_SO_4_ ratios produced severe pitting, demonstrating the strong dependence of corrosion mode on acid speciation and concentration.

In pipeline transport for CCS, CO_2_ is likely to be transported at elevated pressures (typically 40–150 bar) and often near the dense-phase/supercritical region [81,82]. These conditions increase the relevance of impurity-related phase behavior and, therefore, the risk of acid dropout. Dense-phase conditions enhance the solubility of many impurities at steady state, but during transient events such as cooling, temporary depressurization, shutdown, or start-up, rapid crossing of dew-point boundaries leads to the release, concentration, and condensation of impurities. These studies show direct experimental evidence that even small amounts of SO_2_/NO_2_/O_2_/H_2_S/H_2_O in dense-phase CO_2_ can generate H_2_SO_4_/HNO_3_-rich phases with very low pH, causing severe localized corrosion. However, these studies alone cannot explain when and where these acid-rich phases will generate in the presence of actual transient flow conditions. Hence, thermodynamic and kinetic modeling is needed, which is discussed in Section 2.2.

Understanding acid dropout mechanisms is therefore essential for determining CO_2_ purity specifications, developing impurity tolerance limits, selecting corrosion-resistant materials, and designing operational strategies that minimize transient exposure. To compare different dense-phase CO_2_ with key impurities (H_2_O, O_2_, SO_2_, NO_2_, and H_2_S) studies under equivalent conditions, we have summarized CO_2_ phase, temperature, pressure, impurity injection mode and concentrations, corrosion rate, and observed damage in Table 5.

### 2.2. Acid Formation Mechanisms in Impure CO_2_

#### 2.2.1. Primary Reaction Pathways

**Figure 1:**
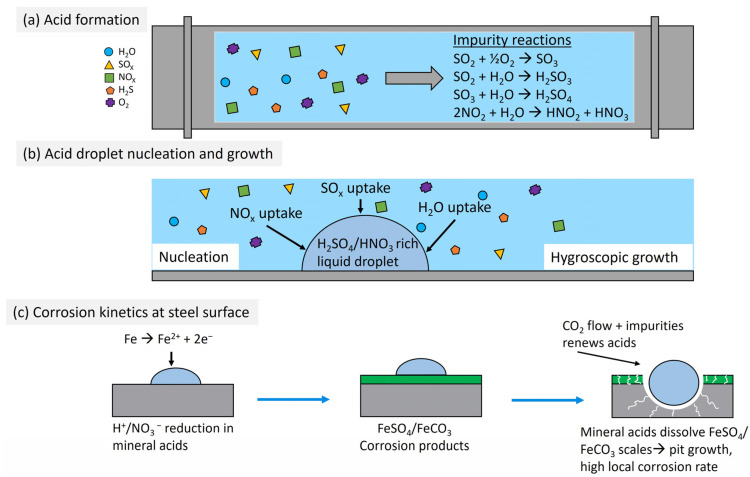
Schematic representation of different stages of acid dropout-based corrosion mechanism.

Acid formation in CCS arises from coupled oxidation, reduction, hydration, and hydrolysis reactions involving sulfur-, nitrogen-, and oxygen-bearing impurities in the presence of trace water, which may be present either as dissolved H_2_O in the CO_2_-rich phase or as separate liquid water. These reactions occur under dynamically changing pressure–temperature (P-T) conditions across capture units, compression trains, transportation pipelines, and injection wells. The overall mechanism consists of three linked stages (Figure 1) [64,87,88]:

(a) Chemical formation of strong inorganic acids (primarily sulfuric acid and nitric acid);
(b) Nucleation and condensation of aqueous acid phases within dense- or supercritical CO_2_;
(c) Interfacial reactions of acid condensates with pipeline materials.

Transported CO_2_ streams typically contain trace amounts of SO_x_ (SO_2_, SO_3_), NO_x_ (NO, NO_2_), H_2_S, O_2_, and H_2_O, depending on the capture process and feedstock. In the dense or gas phase, SO_x_ and NO_x_ impurities undergo a series of oxidation reactions, generating highly reactive intermediates that can form strong acids. NO_2_, being a strong oxidant for SO_2_ and H_2_S, plays a crucial role in that even small amounts of NO_2_ can oxidize SO_2_ to SO_3_, a precursor that forms sulfuric acid in the presence of water. Experimental data for dense CO_2_ at 25 °C and 100 bar show that when SO_2_, NO_2_, O_2_, and H_2_O concentrations were in the few tens of ppm(v) range (~35 ppm(v) SO_2_, NO_2_, H_2_S, and 95 ppm(v) O_2_), a separate liquid phase containing H_2_SO_4_ and HNO_3_ formed. While at lower impurity concentrations (5 and 10 ppm(v)), no corrosive species were observed [89].

Although NO is weakly reactive on its own, it is rapidly converted to NO_2_ when O_2_ is present. Therefore, when O_2_ is present, consideration of total NO_X_ is more important in acid dropout rather than individual NO and NO_2_ concentrations. Because of NO_2_ regeneration in the presence of oxygen, only a trace amount of NO_2_ is required to oxidize H_2_S. NO_2_ decreases only through HNO_3_ formation or corrosion-induced consumption [64,80,90].

Since anhydrous CO_2_ is non-corrosive, the presence of H_2_O is essential for acid formation as it provides a reaction medium for hydration, hydrolysis, and dissolution. H_2_O (either dissolved in the CO_2_-rich phase or as liquid water) converts SO_3_ to H_2_SO_4_ and supports the formation of HNO_3_ from NO_2_ or its oxidized derivatives. The hygroscopic nature of both H_2_SO_4_ and HNO_3_ allows them to extract additional H_2_O from the bulk CO_2_ stream, stabilizing and concentrating the acidic droplets, which then act as microreactors for continued acid growth [69,91,92]. Recent experimental studies in dense-phase CO_2_ have found that the collective presence of SO_2_, NO_2_, O_2_, H_2_S, and trace water leads to far more extensive acid formation than would be predicted from individual impurity behavior, as the interaction among these impurities drops the impurity thresholds required for acid generation [64,69,79,93]. For instance, SO_2_ oxidation proceeds slowly in the absence of NO_2_ but is significantly accelerated by NO_2_ or O_2_. Depending on composition, H_2_S may form elemental sulfur in the presence of O_2_ or H_2_SO_4_ through NO_2_-promoted oxidation.

In a recent paper, Sonke et al. combined experimental studies with chemical-equilibrium calculations to study the correlation between the chemistry of impurities and acid dropout in dense-phase CO_2_ at 100 bar and 4–25 °C [93]. They mentioned that a mixture of H_2_O, SO_2_, H_2_S, NO_2_, and O_2_ may form a separate polar phase of concentrated H_2_SO_4_ and HNO_3_, along with solid sulfur, once a certain mixed-impurity concentration is exceeded. To represent the combined oxidizing and proton-donor capacity of the mixture, they introduced an equivalent sulfuric acid concentration (C_acid_), a scalar indicator of strong acid dropout tendencies.

$$C_{acid}=\left[H_{2}{SO}_{4}\right]+\frac{1}{2}\times \left[{HNO}_{3}\right]+\frac{1}{2}\times [{HNO}_{2}]$$

When sulfur-containing impurities are dominant, the identified critical C_acid_ thresholds to avoid acid participation were 0.5 mM at 25 °C and 0.1–0.2 mM at 4 °C. The experiments confirmed that both H_2_S (proton donor) and NO_2_ (initiator) are needed for the radical chain oxidation to proceed, and increased O_2_ and additional H_2_O both lowered the precipitation threshold. This underlines the correlation between oxidizing strength and hydration level.

The most common impurity interactions are summarized in the table (Table 6), but they represent only simplified overall reactions, as formation of intermediate species such as HSO_3_^−^, HONO, or peroxy-radicals may also take place.

Whether the generated acids remain dissolved in dense-phase CO_2_ or condense as separate aqueous phases depends on their solubility. Studies by Morland and co-workers reveal that H_2_SO_4_ has extremely low solubility under typical pipeline pressures and temperatures, several orders of magnitude lower than nitric acid [79,89,94,95]. However, the solubility of sulfuric acid in dense CO_2_ increases with increasing pressure. It increased from 60 ± 30 ppb mole at 78 bar to 1000–2500 ppb mole at 95 bar or more, due to CO_2_ phase transition from a vapor-like to a significantly denser, liquid-like supercritical state. Nitric acid followed a similar solubility trend but with higher solubility around 80 bar and a shallow minimum around 100 bar. Due to the volatile nature of nitric acid in the gas phase, the mole fraction of nitric acid in the gas phase was ∼4 orders of magnitude higher than that of sulfuric acid. Therefore, H_2_SO_4_ has the tendency to quickly exceed its low solubility limit and condense and accumulate as an acid-rich phase, while HNO_3_ may remain partially dissolved in the CO_2_-rich phase until its concentration reaches its higher solubility limit. Although sulfuric acid is the primary driver of acid dropout, the presence of nitric acid can further increase the corrosivity of condensed liquid by lowering pH and hindering the formation of protective corrosion product films.

#### 2.2.2. Thermodynamic and Phase-Behavior Modeling

Thermodynamic models based on the Mixed-Solvent Electrolyte (MSE) framework that are used commercially (for example, in OLI Systems software (V12)) have been used to predict phase boundaries, water and acid solubilities, and dropout behavior in multi-component CO_2_-rich mixtures [96,97,98]. When compared with dense-phase CO_2_ autoclave experiments, it was found that even though these models capture qualitative data, the predicted impurity concentrations at which the acid formation and precipitation take place are often lower than the observed experimental data.

#### 2.2.3. Kinetic Limitations and Non-Equilibrium Effects

The discrepancy between experimental studies and prediction models is generally ascribed to the kinetic limitations, incomplete mixing, and non-equilibrium phase development in fast-flowing pipeline environments, where residence time and hydrodynamics are different than those in static or semi-static autoclaves, which are factors not captured by equilibrium models [64,89]. Consequently, the impurity limits proposed by experimental studies are more restrictive than those predicted by thermodynamic models. Therefore, acid formation must be evaluated not only using equilibrium solubility but also considering kinetics, water distribution, transient operations (shutdown, depressurization), and hydrodynamic effects.

#### 2.2.4. Mechanistic Speculations

After speculating experimental data, reaction chemistry, and thermodynamic modeling, it can be concluded that NO_2_ initiates the radical oxidation of SO_2_ and H_2_S, which, in the presence of O_2_ and H_2_O, generates acidic H_2_SO_4_/HNO_3_ phases. These initial droplets act as microreactors, supporting further impurity reactions, leading to severe localized corrosion on the steel substrate. Despite this clear understanding, important aspects such as detailed impurity reactions under dense-phase conditions, the nucleation pathways of the initial acid droplets, and the influence of hydrodynamics, wall roughness, and surface chemistry on the spatial distribution of acid dropout in real pipelines and well are still unclear. Future experimental and modeling studies need to translate this qualitative mechanistic context into quantitatively validated predictive tools so that it can be utilized in design and risk assessment in CCS.

### 2.3. Factors Affecting the Acid Dropout

**Figure 2:**
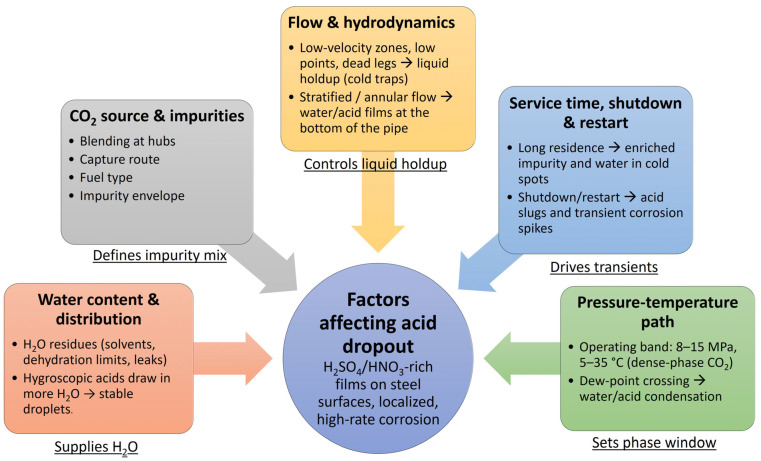
Factors affecting the acid dropout in CCS.

When discussing acid drop-out in CO_2_ transport systems, it is important to recognize that it depends not only on individual impurity concentrations but also on the combined influence of feed composition, thermodynamic trajectory, water availability, flow regime, surface chemistry, and operational transients. These interrelating parameters determine whether (i) the acid-forming reactions can proceed, and (ii) a condensed liquid-phase nucleation can occur; it also determines (iii) the severity of corrosion associated with the resulting acidic films. Understanding these interdependencies is essential for predicting high-risk operating envelopes and designing resilient transport networks (Figure 2).

#### 2.3.1. Source Composition and Impurity Profile

The origin of the CO_2_ stream is a primary determinant that defines the baseline impurity matrix and the intrinsic potential for acid formation. For instance, post-combustion flue gases often contain O_2_, NO_X_, and elevated water, whereas the pre-combustion or gasification streams exhibit higher H_2_S and lower O_2_. Oxy-fuel combustion or industrial emitters (e.g., cement, steel, biomass) may include high SO_X_/NO_X_ with significant moisture.

When CO_2_ streams from multiple sources are blended, impurities that were individually benign can form reactive pairs (e.g., NO_2_ + H_2_S → rapid oxidation pathways leading to H_2_SO_4_) [87,93,99]. For example, Morland et al. conducted a simulated CO_2_ hub study in which they modeled different capture sources with distinct types of impurities. After mixing, the hub system contained low levels of O_2_, SO_2_, H_2_S, NO_2_, and H_2_O (ranging from 5 to 35 ppm(v)) at a total pressure of 100 bar and 25 °C. The results showed that reactions between certain species took place even at concentrations as low as 5 ppm(v), but the reaction products were non-corrosive. However, at impurity concentrations >35 ppm(v), acid dropout and solid formation were observed [87]. Therefore, impurity limits should be defined for the full mixture rather than on an individual-species basis if multiple CO_2_ feed streams are likely to mix in hubs.

#### 2.3.2. Thermodynamic Path: Pressure and Temperature

Thermodynamic conditions, especially the T-P trajectory, along the pipeline control the solubility of impurities, water activity, and onset of multiphase behavior [54,100]. Because of high density and stable single-phase conditions, supercritical CO_2_ (≥7.38 MPa, ≥31.1 °C) is preferred for pipeline transport. In practice, most pipelines operate between 8 and 15 MPa and 5–35 °C. Therefore, even when the pressure remains above the critical value, the local wall temperature may fall below the critical temperature, leading to localized regions of dense liquid CO_2_ along the pipe. The water solubility is maximum, and many impurities remain molecularly dispersed in this dense-phase region. Experimental studies indicate that in this region, acid formation reactions are diffusion-limited and comparatively slow if a condensed liquid phase is absent [101,102]. As the pressure decreases along the pipeline or during operational events, water solubility drops sharply, and this region becomes supersaturated. As soon as the dew-point is crossed, even tens of ppm of water can nucleate interfacial liquid films on steel surfaces. These microfilms act as high-reactivity microreactors with high-reactivity and thus allow for the rapid uptake of SO_2_ and NO_2_ uptake, and the consequently instantaneous formation of H_2_SO_4_ and HNO_3_ [89].

Temperature fluctuations throughout the pipeline also influence the acid dropout. At lower temperatures (<10 °C), CO_2_ density increases, and impurity solubilities shift toward phase separation, particularly for SO_2_-H_2_O mixtures. Hua et al. studied the effect of temperature and water content on the corrosion response of X65 steel in supercritical CO_2_ environments. For similar H_2_O concentrations at 35 °C and 50 °C (both at 8 MPa), they found higher general corrosion rates and more severe localized attack at 35 °C, due to the higher CO_2_ density and less protective surface films at this temperature. At 50 °C, the corrosion products were denser and more protective [103]. At low temperatures, the volatility of nitric species is also suppressed, favoring NO_2_ uptake into the growing aqueous phase [80]. In a recent study, Simonson et al. investigated the interaction between temperature and H_2_O concentrations and their effects on the corrosion behavior of X52 and GR70 steel. They found that in the presence of NO_2_, at 70 ppm(v) H_2_O concentration, the corrosion rate was five and three times greater for X52 and GR70 steel, respectively, at 5 °C compared to 25 °C [80]. The temperature gradients close to compressor stations or solar-heated above-ground parts may temporarily increase water solubility, delaying condensation until the CO_2_ cools again downstream [102].

Since different acid species respond very differently to pressure, the influence of pressure on the system during the condensation of acids must also be considered. Experimental studies show that nitric acid has comparatively high solubility in dense-phase CO_2_, and that this solubility increases markedly with pressure. While sulfuric acid is essentially insoluble in the dense phase, H_2_SO_4_ preferentially nucleates and accumulates as a separate liquid even under otherwise high-pressure conditions. Since water solubility in CO_2_ is strongly pressure-dependent, pressure transients are high-risk events, ascribed to the reduced solubility and (through Joule–Thomson cooling) temperature fall. This compounds the super-saturation and acceleration of the formation of acids and their dropout [101]. For instance, Xu et al. investigated the corrosion behavior of supercritical CO_2_/SO_2_/O_2_ in the presence of water on X60, X65, X70, and X80 carbon steels at 50 °C and 8–12 MPa [104]. They found that, due to changes in pressure, the solubility of H_2_O in CO_2_ also changed, and hence the corrosion rate was affected. The degrees of general and localized corrosion were higher at 8 MPa than at 10 MPa, with water contents ranging from 1600 ppm to 2600 ppm, but lower at 3000 ppm. This could be because of the condensation of a thick liquid water phase and associated changes in mass transport and film protectiveness.

Since rapid depressurization can cool the fluid by 20–50 °C within seconds due to operational venting or emergency shutdown, it causes water condensation (creating conditions favorable for acid formation even if the bulk stream remains conceptually dry), followed by the precipitation of hydrates, dry ice, or mixed-acid droplets [75]. Therefore, mapping the pipeline’s thermodynamic path (including compressor stations, coolers, elevation changes, and dead legs) along with predicted acid dew-point curves is essential for identifying sections most susceptible to acid dropout.

#### 2.3.3. Mixing and Flow Regime

While thermodynamic conditions determine the equilibrium state of impurity dissolution, flow hydrodynamics influences where and how fast reactive species nucleate droplets, or accumulate at pipeline surfaces [105,106,107]. It is commonly assumed that impurities in CO_2_ streams are uniformly mixed; however, they can exhibit a non-uniform radial and axial manner due to differences in diffusivity, density, and solubility.

Computational fluid-dynamics (CFD) models and field-scale analyses consistently show that liquid phases tend to collect in low-velocity regions, boundary layers, or bottom-of-pipe pockets in stratified or partial two-phase flow, creating localized zones with significantly higher water and impurity activity (i.e., wall films and cold-traps) than the overall bulk [108,109]. When water microdroplets form, their interfaces become sites of accelerated mass transfer, leading to the rapid uptake of SO_2_, NO_2_, and H_2_S. The influence of local mixing intensity, which is set by turbulent eddy scales, wall shear, and the bulk flow regime, is greater on droplet composition and corrosivity than on bulk impurity concentrations. In poorly mixed pockets, sub-threshold impurity concentrations that are safe according to equilibrium predictions can transform into high-acidity microenvironments.

The flow regime is responsible for the distribution of impurities and also affects acid stability. A high flow rate (i.e., turbulent flow) can promote the uniform dispersion of impurities and suppress their localized accumulation. While stratified, annular, or laminar-flow pockets can generate stagnant microenvironments, responsible for the early formation of aqueous films or droplets [108]. Experimental investigations support this behavior. Farelas et al. examined the influence of flow on the corrosion of pipeline steel at 8 MPa under both liquid (25 °C) and supercritical (50 °C) CO_2_, with 650 ppm water and 0.1% SO_2_. The transition from static to dynamic conditions reduced general corrosion from 0.03 to 0.02 mm/y in supercritical CO_2_ and from 0.10 to 0.01 mm/y in liquid CO_2_ [110]. This happened because the presence of flow reduced the level of water accumulation on the steel surface, leading to reduced corrosion rate in the system as compared to the static conditions [111].

However, the effect of flow is not always protective. High flow rates can increase the availability of corrosive ions to the metal surface, which accelerates the corrosion process. The increased flow rate might carry Fe^2+^ ions away from the metal surface, making it less likely to form a stable protective FeCO_3_ film [105,112]. For instance, Wei et al. studied the corrosion behavior of X70 steel in CO_2_-saturated water under both static and dynamic conditions with different flow rates ranging from 0 to 2 m/s. They found that variations in flow rate changed the corrosion type from uniform to localized [106]. Under dynamic conditions, the corrosion rate and pit depth were higher than under static conditions, attributed to the absence of corrosion products (FeCO_3_), which allowed the corrosive medium to more easily reach the metal surface, leading to severe localized corrosion. Under static conditions, the number and size of FeCO_3_ particles increased over time, leading to a thicker, denser FeCO_3_ corrosion product layer and, hence, uniform corrosion over time.

It should be noted that other studies have reported insignificant dependence on flow rate under certain conditions [113]. Silva da Sá et al. examined API 5L X80 steel in water-saturated supercritical CO_2_ at 35 °C and 80 bar and observed no significant variation in corrosion rate across flow velocities of 0.15–1 m/s [114]. They mentioned that the lack of dependence was due to the extremely small droplet size, which remained below the threshold required for hydrodynamic forces to alter droplet distribution or surface wetting; therefore, flow effects are negligible.

As shown in these results, the influence of flow on acid drop-out is non-linear and regime-dependent. An investigation into the effects of impurity chemistry, droplet dynamics, local hydrodynamics, and corrosion-film stability determines the flow behavior in protective or detrimental ways.

#### 2.3.4. Service Time, Shutdown, and Accumulation Dynamics

The duration of water residence, impurity accumulation, and interfacial exposure to corrosive species is strongly influenced by service time and operational downtime. Experimental and modeling studies suggest that the presence of impurities such as SO_2_, NO_x_, and H_2_O-bearing species in stagnant or low-velocity zones can cause them to accumulate in condensed liquid films on the pipe surface. This will increase local concentrations to levels much higher than the bulk CO_2_ composition, even when bulk impurity levels are only in the low-ppm range [115,116].

Intentional shutdown events, such as maintenance and operational schedules, or unintentional shutdown events, such as the loss of compression and trip, can accelerate these processes. CO_2_ shows significantly different shut-in behavior compared to water and hydrocarbons due to its strong pressure–temperature dependence and outstanding Joule–Thomson effects, which lead to the possibility of two-phase flow [117]. Because of temperature and pressure drop, two-phase becomes favorable, ascribed to the vapor–liquid equilibria shift, leading to the promoted condensation of H_2_O, NO_2_, SO_2_, and hydrate or liquid water films [118]. According to the PHMSA (Pipeline and Hazardous Materials Safety Administration) database, an average pipeline shutdown duration is approximately 53 h [119,120].

During prolonged shutdown/static periods, impurities slowly migrate towards low-temperature zones due to natural convection and give rise to cold-traps, where water activity increases and rapid acid formation is enabled. Various autoclave and loop-testing studies show that, even when only small amounts of liquid water have condensed (corresponding to bulk H_2_O levels of <300 ppm), bisulfite/sulfite chemistry and NO_2_ hydrolysis in wall films can proceed rapidly during cool-down. Hence, H_2_SO_4_- and HNO_3_-rich liquids are formed within hours to days of shutdown, depending on impurity composition and the local temperature profile [64,69,79,115,121,122,123].

Restarting the flow after shutdown carries its own risks because restart conditions can induce the shear-driven detachment of accumulated acid films or droplets, leading to their redistribution along downstream pipeline sections. Usually, the initial slug contains a highly concentrated mixture of acids, dissolved iron, and water, leading to transient spikes in corrosion rate. Thus, shutdown exposure introduces a complex interaction of condensation, impurity enrichment, interfacial chemistry, and restart hydrodynamics. They together significantly increase the acid dropout risk. Therefore, long-term service consistency involves not only impurity control and thermodynamic assessment but also careful considerations of shutdown conditions, drainage design, and post-shutdown flushing.

## 3. Impacts of Acid Dropout

There are several ways acid dropout can influence CCS operations, from pipeline design and operation to geological and storage considerations. The interactions of trace impurities (SO_2_, NO_2_, O_2_, H_2_S) with condensed water under specific thermodynamic and hydrodynamic conditions give rise to the formation of highly acidic phases, which alter the physical behavior of the CO_2_ stream, ultimately inducing critical chemical degradation of pipeline materials [90]. The impact of acid dropout can be categorized as an impact on thermodynamic properties and a chemical impact that is basically determined by corrosive condensates such as H_2_SO_4_ and HNO_3_ [124]. Since the understanding of these effects is critical for designing impurity specifications, predictive models, and mitigation strategies, Section 3 discusses the physical and chemical impacts of acid dropout on the CCS system.

### 3.1. Impact on Thermodynamic Properties

The presence of condensable, acid-forming impurities (SO_2_, NO_2_, H_2_S, and reactive O_2_) significantly modifies the thermodynamic behavior of CO_2_ mixtures through changing phase equilibria, solubility limits, and interfacial stability under transport and storage conditions [125,126]. Compared with non-condensable impurities, acid-forming impurities participate in strong intermolecular interactions and chemical reactions with water, which affect their respective thermodynamic behavior. The CO_2_ phase envelope distorts in the presence of SO_2_, NO_2_, H_2_S, and O_2_ due to a decrease in the mixture critical point, shift in the dew and bubble curves, and the expansion of the two-phase region as compared to the pure CO_2_ system [127,128,129]. Consequently, condensation and phase separation can occur at higher temperatures and pressures than predicted by binary CO_2_-water equilibria; hence, the practical safe operating window for single-phase dense CO_2_ transport is narrower than that shown in simple CO_2_-H_2_O design charts.

In impurity-driven reactions and acid formation, the CO_2_ stream transitions from a homogeneous, dense fluid to a heterogeneous multiphase system containing bulk CO_2_ and dispersed or wall-based acidic liquid phases. The formation of H_2_SO_4_ and HNO_3_ through in situ reactions significantly increases water activity coefficients and reduces CO_2_ solubility in these condensed acid-rich liquid phases, promoting the persistent formation of liquid droplets [72]. Since both acids are extremely non-volatile and highly polar, they favorably partition into the liquid phase, reducing the effective vapor pressure of water and increasing nonuniformity in the surrounding CO_2_ phase. This behavior promotes liquid–liquid equilibria (LLE) between acid-rich liquid droplets and the CO_2_-rich phase. This phenomenon is not captured by conventional vapor–liquid equilibrium (VLE) models that are commonly used for pipeline design [96,130].

**Figure 3:**
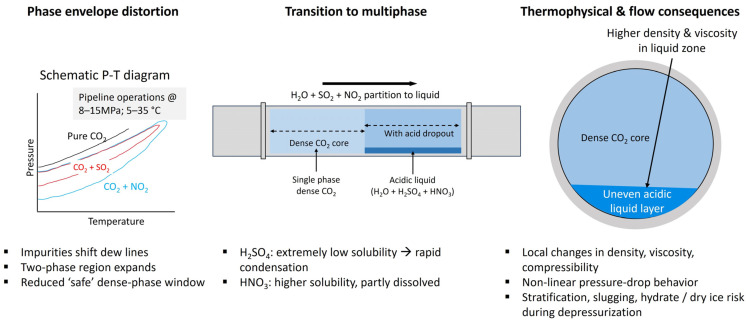
Schematic representation of the impact of acid dropout on the thermodynamic properties of the CO_2_ mixture system. P-T data in the schematic diagram are taken from [132].

The presence of any significant acid droplet levels can cause measurable changes in local thermophysical properties, including density, viscosity, and compressibility. These local changes give rise to non-linear shifts in pressure-drop behavior, increased frictional losses, and enhanced susceptibility to flow stratification and intermittent slugging [63,80,131]. Because of their higher density compared to CO_2_, acidic liquid films preferentially deposit at the bottom of the pipe, leading to uneven liquid holdup and increased thermal gradients. The Joule–Thompson cooling of the CO_2_ mixture during depressurization or throttling, combined with additional cooling from the flashing or evaporation of the accumulated liquid, can result in localized cooling and an increased risk of secondary phase transitions, such as dry ice formation or hydrate nucleation. Consequently, this may induce flow instability and operational shutdowns. Figure 3 represents the impact of acid dropout on the thermodynamic properties of the CO_2_ mixture system.

In subsurface storage, the higher density and viscosity of acid-rich liquid phases, compared with CO_2_, promote gravitational segregation and localized accumulation within pore spaces or near wellbores. Because of this, the effective permeability of the region is modified, which may reduce CO_2_ injectivity and increase rock–fluid and fluid–metal interactions [63]. In all, the thermodynamic impact of condensable impurities ranges beyond the concept of simple phase separation, introducing combined effects of non-ideal mixing, reactive phase formation, and dynamic multiphase behavior, which fundamentally challenge the conventional assumptions used in CO_2_ transport and storage design [80,133].

### 3.2. Chemical Impact

Because of acid dropout, very aggressive, localized acid-rich liquid regions form within otherwise nominally dry, dense CO_2_ streams (i.e., no bulk free water but only dissolved H_2_O). The chemical impacts are governed by the formation, enrichment, and persistence of sulfuric (H_2_SO_4_) and nitric (HNO_3_) acid phases, which lead to rapid metal dissolution, changes in corrosion product chemistry, and degradation risks in carbon capture and transport systems [67,77,134]. Conventional wet-CO_2_ corrosion of carbon steel in oil and gas and CO_2_-EOR service is relatively well-understood and predictable, whereas corrosion due to acid dropout in CCS is governed by complex thermodynamic, kinetic, and transport-controlled processes that are not adequately captured by current testing methodologies and models. Hence, chemical attack is influenced not only by impurity concentrations but also by phase behavior, liquid accumulation, replenishment dynamics, and local chemistry at the metal interface.

#### 3.2.1. Initiation and Evolution of Corrosive Acidic Phases

The chemical impact of acid dropout begins with the nucleation of a separate acid-rich liquid phase within a dense CO_2_ liquid or gas phase. Nucleation is usually triggered at metal surfaces or pre-existing defects such as corrosion products, inclusions, or deposits, and reactive impurities such as SO_2_, NO_2_, O_2_, and H_2_S accelerate this process via in situ chemical reactions, producing strong acids [64,96,135]. Practically, when the local solubility limits of acids in the CO_2_ are exceeded, a low-pH liquid phase can nucleate at the wall surface, even though the bulk composition of the stream remains below the solubility limits of acids in CO_2_. Currently, the capabilities of predictive thermodynamic models are limited by the lack of equations of state (EOS) that can describe multi-component CO_2_ systems at very low impurity concentrations and the associated chemical reactions [136].

After nucleation, the liquid phase grows directly on the pipe wall by drawing in more water and other polar species from the surrounding CO_2_, leading to corrosion below the predicted solubility thresholds. The composition of the resulting liquid continues to change due to dilution by water, continued acid formation, and interactions with corrosion products. It is difficult to design equilibrium-based predictive models because, even when bulk CO_2_ specifications are within limits to prevent acid dropout, local microenvironments can still lead to acid formation and retention.

#### 3.2.2. Dropout, Accumulation, and Replenishment Effects

Depending on the flow conditions, dropout acids form a constant corrosive environment; the rate of condensation and further collection of acidic liquids, especially at low points, weld areas, or stagnant regions of the pipeline, amplifies the severity of the chemical impact of acid dropout [137,138].

Replenishment of corrodents is a critical but often overlooked factor that strongly influences corrosion rate [139]. In closed laboratory systems (autoclave corrosion testing) without replenishment, the actual corrosion rate will be underestimated because the consumption of corrodents increases pH and reduces the corrosion rate as the environment becomes less aggressive. In contrast, field conditions allow continuous replenishment of corrosive species, resulting in sustained high corrosion rates that are inconsistent with impurity levels measured in the bulk stream.

#### 3.2.3. Corrosion Kinetics Due to Acid Dropout

Once a corrosive acidic phase is established, corrosion is initiated by the electrochemical dissolution of iron, generating aqueous Fe^+2^ and consuming H+, thereby increasing the local pH. This may temporarily slow corrosion and promote the precipitation of corrosion products. Under conditions dominated by carbonic acid, FeCO_3_ can form protective scales if super-saturation and kinetics are favorable. However, in acid dropout environments, where H_2_SO_4_ and HNO_3_ are formed, the predominant solid phases are iron (II/III) sulfates and nitrates (such as FeSO_4_, Fe_2_(SO_4_)_3_, FE(NO_3_)_2_, Fe(NO_3_)_3_). When H_2_SO_4_ is formed, the formation of iron sulfate scales takes place, which may provide partial protection under static conditions but they are prone to breakdown under flow, giving rise to severe localized corrosion [69,140]. The factors that influence this process are water content, movement (velocity, agitation, and replenishment), and temperature. In case of HNO_3_ dropout, corrosion is further aggravated, either as a primary dropout species or formed within an existing aqueous phase, although HNO_3_ is less likely to precipitate as a pure phase [80,141]. Under continuous acid exposure, various studies have reported extreme localized corrosion rates, including deep pitting. The type of corrosion is influenced by the integrity and stability of corrosion product films, the acidity and composition of the liquid phase, and hydrodynamic conditions [90,142]. When the protective scale breaks down, a higher corrosion/penetration rate is predicted, which is way higher than that from a uniform corrosion mechanism. Table 7 presents examples of corrosion tests on pipeline steel in dense/supercritical CO_2_ containing acid-forming impurities, indicating the roles of SO_2_, NO_2_, and related species on corrosion rate and film composition.

#### 3.2.4. Effect of Flow on Local Corrosion Chemistry

It was discussed in Section 2.3.3 that the flow redistributes the acid-rich phases along the pipe. It also affects the local chemistry at the metal–solution interface by changing mass-transfer rates, acid-rich film residence time, and the stability of corrosion products. From long-term flow-loop and rotating-cage studies, it was found that in dense/supercritical CO_2_, with increasing flow velocity, the supply of H^+^, SO_4_^2−^, NO_3_^−^, and other species to the steel surface also increases. This initiates the removal or destabilization of FeCO_3_ and Fe sulfate/nitrate films, leading to severe localized corrosion under dynamic conditions. On the other hand, in mist (refers to droplet nucleation) systems, where droplet size is very small for hydrodynamic detachment, there is a negligible effect of flow on the corrosion. In all, the effect of flow on acid dropout is a regime-dependent phenomenon.

Corrosion due to water dropout (or conventional CO_2_ corrosion), where corrosion is caused by carbonic acid, is well-understood as compared to the corrosion due to acid dropout [146]. When water is dissolved in CO_2_, corrosion does not take place. The condensation of water on the metal substrate leads to CO_2_ corrosion. Although alcohols and glycols, including methanol, monoethylene glycol (MEG), and diethylene glycol (DEG), may delay water dropout or inhibit corrosion, their usefulness is critically influenced by concentration, phase, and interactions with other impurities.

Numerous experimental studies have examined the corrosivity of acid dropout, but reported corrosion rates are lower than actual rates because test conditions account only for corrosion in the bulk phase and do not include the corrosive effects of accumulated liquids at low points. In addition, there are many other parameters, including temperature decrease, depressurization, the presence of a new unknown impurity, a temporary increase in some impurities, accidental ingress of species, and the presence of catalytic species, that will be responsible for acid dropout.

It can be concluded that the corrosion severity during acid drop-out depends on the evolving composition of condensed liquids rather than on bulk impurity concentration alone, highlighting the need for careful impurity specifications, dynamic monitoring, and mitigation strategies customized to liquid-phase chemistry rather than to gas-phase chemistry alone.

## 4. Mitigation Strategies for Chemical Degradation and Acid Dropout

**Figure 4:**
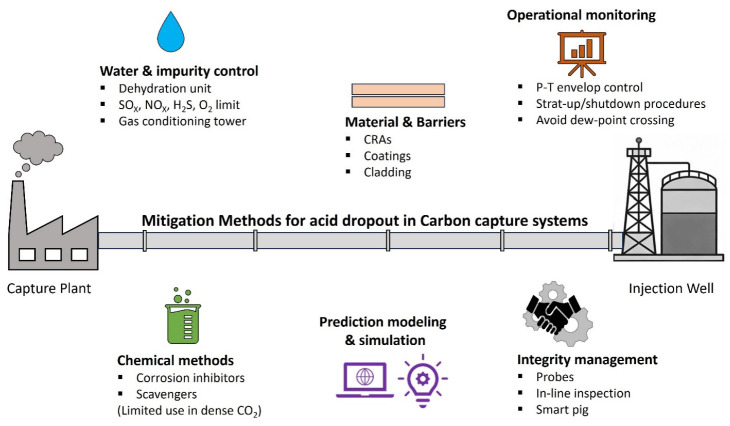
Mitigation strategies for acid dropout in carbon capture plants and transport systems.

To mitigate the corrosion caused by chemical reactions and acid dropout, a combination of multiple approaches, including converging impurity control, materials selection, process design, chemical treatment, and operational monitoring, is needed (Figure 4). Since different degradation mechanisms initiate only under specific liquid dropout conditions, countermeasures must be validated across the full operating window, including upset, transient, and low-temperature conditions.

Currently, only a few mitigation strategies can be considered well-established for dense-phase CO_2_ with impurities, while others are at the laboratory demonstration or conceptual stages. Dehydration, along with strict impurity limits and safe operating windows, is the only strategy that is widely implemented and proven for preventing acid dropout corrosion in carbon steel in CCS systems. Chemical inhibition, advanced coatings, and some CRA and hybrid solutions have so far been tested in laboratories or in oil and gas environments. There is still very little long-term field data available on their performance in a dense CO_2_ stream with realistic multi-impurity profiles.

### 4.1. Control of Water Content, Phase Stability, and Impurity Specification

The most important strategy, and at present the only proven strategy, for the corrosion control of carbon steel infrastructure in CO_2_ transport systems is to dehydrate the system (maintain water content below the dew-point) to prevent free water or acid condensation across all normal operating windows. However, as we mentioned earlier, acid formation and dropout can occur via chemical pathways that are difficult to predict solely from gas-phase considerations. From various experimental and modeling studies, it was found that corrosion rates in dense-phase CO_2_ remain negligible when free water and reactive impurities such as SO_2_, NO_2_, O_2_, and H_2_S are kept below system-specific thresholds, while even small changes in water or impurity levels can initiate the formation of highly corrosive H_2_SO_4_/HNO_3_-rich condensates, leading to rapid material degradation [83,87,147]. From the experimental studies where multiple impurities were present, it was found that, in the presence of SO_2_, NO_2,_ and O_2_, water contents that would be safe in a binary system can give rise to an acid-rich condensate with a pH near or below two, leading to high corrosion rates even at sub-saturation water levels [72]. Therefore, water control cannot be considered independently of impurity composition, as the acceptable limit for water depends on the concentrations and ratios of acid precursors and oxidants present in the CO_2_ stream. Although industries currently use deep dehydration along with typical impurity limits as the primary proven basis for corrosion control, they still rely on a relatively small number of mixed-impurity datasets. To address this, recent IEAGHG guidance emphasizes that deep dehydration should be the minimum requirement, and in those cases where impurity levels reach the conditions that can form strong acid condensates, even stricter limits are required [148].

Ideally, a transported CO_2_ stream should be maintained within a single, well-defined thermodynamic phase, avoiding local temperature–pressure points that are thermodynamically favorable for the formation and precipitation of water-rich or acid-rich liquids. Transient events such as depressurization, Joule–Thomson cooling at valves, start-ups, and shutdowns can lead to localized cooling and pressure drop, shifting the system into regions where the solubility of water and acids is reduced, and condensation is favored [74,139,149]. Consequently, it is essential to design operating envelopes that keep the CO_2_ stream within the desired single-phase region under all predictable conditions, carefully control pressure profiles along the pipeline, and implement conventional limits on minimum metal temperatures at constrictions and control valves.

The CCS hub projects have the potential to meet the decarbonization needs of various industries, including both large and small emitters. Since impurities may come from different reactors, burners, solvent-based processes, etc., depending on the project, it is important to have a full understanding of acid formation and precipitation thresholds. Recently, Sonke et al. proposed control strategies for the formation and precipitation of acid for a CCS hub [93]: (i) controlling total sulfur and nitrogen to limit the maximum amount of formed strong acids; (ii) controlling the oxidizing strength (O_2_, NO_2_) so that completion of radical chains can be prevented; (iii) controlling all potential H atom sources that would hydrate sulfur and nitrogen oxides to form acids (not just H_2_O, as usually considered). These approaches are consistent with the more general idea of impurity envelopes rather than single-impurity limits.

The most critical countermeasure is to define and apply upper impurity limits, especially for species more likely to participate in acid-forming reactions, such as SO_X_, NO_X_, O_2_, H_2_S, and H_2_O. Because of its radical nature and high reactivity, NO_2_ is the main concern. NO_2_ promotes HNO_3_ formation even at very low concentrations and in the presence of H_2_O. This is also reflected in specifications such as the Northern Lights liquid CO_2_ standard that limits NO_2_ to 1.5 ppm(mol) [62,150,151]. It was found that in the presence of NO_2_, the allowed impurity threshold for species that are otherwise benign reduces because the reaction kinetics accelerate and co-condensation occurs [69,96]. Hence, upper impurity limits should be defined based on their acid formation potential, solubility limits in dense and gas-phase CO_2_, and combined impurity effects instead of single-component thresholds [84]. These impurity specifications must be validated for temperature and pressure transients, start-up, shutdown, and various flow conditions [123]. Additionally, various CO_2_ stream mixtures must be accounted for, as they may decrease solubility limits and lead to premature liquid dropout.

### 4.2. Chemical Inhibition Strategies

Chemical countermeasures can be used to either reduce corrosivity or interfere with acid-forming reactions.

Corrosion inhibitors: Chemical inhibition is also being explored for CO_2_ systems, where the same concepts used for oil and gas are applied, but with the understanding that traditional inhibitor chemistries may behave differently in dense-phase CO_2_ and mixed water–acid droplets. The most important criteria for an effective corrosion inhibitor are that they must be soluble in CO_2_, adsorb strongly onto steel in CO_2_-saturated, low-pH aqueous films and mixed water–acid droplets, and be stable in the presence of oxidizing impurities (O_2_, NO_X_). Imidazolines, amines, and quaternary ammonium/imidazolium salts are the most popular corrosion inhibitors capable of providing protection in CO_2_-rich, low-pH aqueous environments [152,153]. For instance, in a recent study, the corrosion-inhibition effects of imidazoline and piperazine on N80 steel in the water-saturated supercritical CO_2_ phase and supercritical CO_2_-saturated aqueous phase containing impurities (SO_2_, NO_2_, and O_2_) were investigated [154]. It was found that increasing piperazine concentration from 300 ppm to 1000 ppm increased inhibition efficiency from 64% to 86%, attributed to the higher pH of the aqueous phase, which neutralizes acid gas impurities through its imino group and acts as a pH stabilizer. In contrast, imidazoline was less effective, and severe localized attacks were observed in the supercritical CO_2_ and aqueous phases. In a recent review paper, synergistic corrosion mechanisms in supercritical CO_2_ are discussed, and mitigation strategies in environments containing SO_2_ and O_2_ are also mentioned, indicating that chemical inhibition is a key tool alongside impurity control [90]. For SO_2_-containing systems, reducing inhibitors such as thiosulfate, which can react with SO_2_ to form less aggressive species or protective films, and amine-based inhibitors, which partially neutralize sulfurous acid and provide a film-forming barrier, are considered. It is also mentioned that composite inhibitors (e.g., imidazoline plus an inorganic species such as molybdate), combined with pH adjustment of the aqueous phase, can provide more stable protection under fluctuating SO_2_ levels. However, most of the available inhibitor data on CCS-related conditions is based on short-term laboratory testing with simple geometries, and there is no long-term field evidence of successful continuous corrosion inhibition in dense-phase CO_2_ pipelines or hubs transporting mixed-impurity streams. Therefore, the performance of inhibitors under realistic SO_x_/NO_x_/O_2_/H_2_S combinations, very low water contents, and highly acidifying transients is highly uncertain. Currently, these chemistries can be best described as experimental research tools rather than a stand-alone, fully developed mitigation strategy.

Scavengers are designed to remove reactive species such as SO_X_, H_2_S, or NO_X_ before they participate in corrosive chemistry, indirectly preventing acid formation [155,156]. However, their practical application in dry (i.e., no liquid water) CO_2_ systems is constrained by formulation and phase-behavior issues. Most of the commercially used H_2_S scavengers in gas treatment (e.g., triazines, aldehydes, oxazolidines) are water-based or otherwise polar liquid formulations, which are fundamentally incompatible with CO_2_ pipelines that are strictly dehydrated to very low water contents, typically specified via strict dew-point limits (typically, dew-points below −40 °C at operating pressure). Adding such formulations would introduce liquid water and polar components into the system, contradicting the intent of strict dehydration. Hence, scavenger technologies are well-established in conventional gas treatment and upstream applications, but their use in strictly dehydrated, dense-phase CO_2_ transport with multiple reactive impurities has not yet been confirmed beyond conceptual proposals.

Despite promising laboratory results, the practical use of chemical inhibitors and scavengers in CO_2_ transport systems remains in its early stages. There is almost no field data regarding their performance in dense-phase CO_2_ pipelines designed for CCS, particularly under realistic multi-impurity and acid dropout conditions. Other key challenges in the implementation of corrosion inhibitors are their compatibility with capture solvents, dehydration systems, and downstream storage operations, and also the prevention of foam, emulsions, or formation damage [152]. At present, it would be more practical to view chemical inhibition as a local, supplementary measure for high-risk zones (e.g., low points, dead legs, injection wellheads) where modeling and monitoring indicate that recurring wetting and acid accumulation can occur.

### 4.3. Material Selection

Material selection can help to control the effects of localized acid dropout when impurity control alone cannot guarantee its absence [150,151]. Since impurities may initiate localized corrosion, cracking, or rapid metal loss, mitigation should include selecting materials resistant to the worst-case impurity concentrations. Conventionally, carbon steel is used for CO_2_ transportation because it is suitable for well-controlled, dry CO_2,_ given its cost and established experience. However, in CCS-relevant environments, where impurity-driven reactions lead to the formation of H_2_SO_4_ and HNO_3_, usually in the dilute-to-intermediate concentration range, carbon steel corrodes rapidly. Highly concentrated H_2_SO_4_ (>90 wt.%) can be relatively benign for carbon steel, while concentrations <70 wt.% are highly aggressive. Consequently, for CCS networks, where involuntary wetting, transient water breakthrough, or off-specification impurities are potential issues, relying on unlined carbon steel alone is considered high risk, and additional barriers (coatings or corrosion-resistant alloys (CRAs)) are recommended in specific sections [150,157,158].

Appropriate CRA selection can significantly reduce the corrosion risk associated with transporting impure CO_2_. For instance, the incorporation of aluminum into low-Cr steel helped form a dense corrosion product layer, significantly reducing local corrosion sensitivity [159]. Because of the ability to form stable Cr-rich passive films, CRAs such as 13Cr martensitic stainless, 22–25Cr duplex stainless steels, and Ni-based alloys are widely considered more tolerant to wet CO_2_ and acidifying impurities [160,161]. However, the suitability of 13Cr should be tested cautiously in the presence of mineral acids, as it can perform acceptably in wet CO_2_ service, but under strong acidic dropout conditions, resistance may decline substantially. However, high-grade steels such as 316 L or those with 18–21% Cr show significantly lower corrosion rates and better passivation in sulfuric acid media than carbon steel, so they could be more suitable for CO_2_ environments with H_2_SO_4_/HNO_3_-rich condensates. Systematic data describing CRA performance in dense or supercritical CO_2_ with multi-impurities are still emerging. Various autoclave studies have provided quantitative information on general and localized corrosion of super 13Cr martensitic stainless steels, duplex (22Cr), and super-duplex (25Cr) stainless steels in CO_2_, with specific levels of O_2_, SO_2_, NO_2_, H_2_S, and chloride. These studies have been used to develop preliminary material selection charts for downhole and transport applications, showing clear applicability envelopes. CRA-based mitigation has been proven effective for certain envelopes, but its effectiveness is uncertain when applied to new impurity combinations and an over long service life. Table 8 summarizes the different CRA types considered for CCS applications in which acid dropout cannot be fully excluded.

The effectiveness of CRAs is influenced by the levels and types of impurities in the system and by their effects on the pH of the liquid acidic phase. Zhang et al. investigated the corrosion of carbon steel and Cr-containing steel exposed for 96 h in water saturated with supercritical CO_2_ containing controlled amounts of O_2_, CO, and H_2_S impurities [170]. The results showed that CO had almost no influence, whereas increasing O_2_ or H_2_S increased the corrosion rate of carbon steel. However, the rate decreased slightly at higher O_2_ concentrations but still increased with H_2_S in Cr-containing steel. The tests were conducted for only 96 h, which is a short duration for assessing the effect of impurities on the localized corrosion of CRAs. Similarly, Hashizume and their co-workers investigated the performance of 13Cr and Super 13Cr (S13Cr) in SC-CO_2_ [171]. The tests were performed in the absence of O_2_ at 100 °C in a solution containing 30,000 ppm chlorides, at different CO_2_ pressures. It was found that the corrosion rates for 13Cr were 0.07–0.16 mm/y at 300 and 150 bar, respectively. For S13Cr, no localized corrosion was found within the same pressure range, except at 250 bar, where the corrosion rate was 0.01 mm/y. It was observed that both 13Cr and S13Cr alloys were not fully corrosion-resistant and exhibited crevice attack in almost all environments. Matsuo et al. examined Super 13Cr (S13Cr) and 25Cr super-duplex stainless steel (SDSS) in SC-CO_2_ with SO_2_ and O_2_, and found that S13Cr was corrosion resistant in the absence of impurities [165]. For all O_2_ and SO_2_ concentrations tested, S13Cr was unsuitable, whereas the 25Cr SDSS was corrosion-resistant. Based on previous studies, 13Cr should not be used in SC-CO_2_ service with O_2_.

Since the performance of CRAs in dense CO_2_ streams with impurities depends on the collective effects of present impurities and their concentration, chloride concentration, and temperature, it is important to summarize the conditions under which each CRA type has been reported to be reliable. Kanki and their research group have conducted various corrosion studies on 13Cr/super 13Cr martensitic steels and 22–25Cr duplex and superduplex stainless steels in CO_2_ with O_2_, SO_2_, NO_2_, and H_2_S, providing applicability envelopes for CRAs in acidified CO_2_ [163,164,165,172]. In one such study, general corrosion, pitting, and crevice corrosion behavior of super 13Cr (UNS S41426), duplex (UNS S82551), and super-duplex (UNS S39274) alloys were studied at 100–150 °C and 125–300 bar in 5 and 25 wt.% NaCl solutions saturated with CO_2_ containing oxidizing impurities [172]. They reported that 13Cr is generally unsuitable for CCS applications due to its limited corrosion resistance. Super martensitic stainless steel (SMSS, e.g., S41426) can be applicable in a CO_2_ environment when impurity levels are extremely low (as defined by ISO-27913), and chloride concentrations are moderate (up to 5 wt.% or 31,300 mg/L). However, at higher chloride concentrations (~25 wt.% or 181,000 mg/L), 13Cr and SMSS are not recommended because they are highly susceptible to pitting and crevice corrosion, regardless of the presence of oxygen. It can be concluded that for 13Cr and SMSS, the application window in CCS environments is very narrow. Under mixed-impurity conditions, SMSS is not a robust choice, and its use is restricted to mildly acidified, low-chloride, and low-impurity regimes.

Duplex stainless steel (DSS, e.g., S82551) and super-duplex stainless steel (SDSS, e.g., S39274) offer a broader range of applicability but are also affected by chloride content and by oxidizing sulfur- and nitrogen-containing species. From autoclave tests in supercritical CO_2_ at 150 °C with NaCl at 5 and 25 wt.%, it was found that DSS and SDSS both exhibited low corrosion rates and no localized attack in the presence of up to 100 ppm SO_2_. The presence of 100 ppm NO_2_ initiated crevice corrosion in DSS, whereas NO_2_, along with O_2_, led to crevice attack in SDSS at high chloride levels. Therefore, DSS can be a cost-effective solution in low-salinity environments with strictly controlled O_2_, SO_2_, and NO_2_, whereas in higher-salinity or higher-impurity cases, only SDSS consistently exhibits superior resistance to localized corrosion, thereby validating its suitability for downhole CCS applications.

In addition to base-metal performance, several studies have highlighted the critical role of welds and weld overlays in CCS supercritical CO_2_/H_2_S environments. Tests on welded 13% Cr martensitic stainless steel and welded API 5L X65 pipeline steel in supercritical CO_2_ with H_2_S show that the welded region can exhibit accelerated corrosion and cracking under applied stress relative to the parent material [173,174,175]. This shows the need for qualifying weld procedures and H_2_S limits specifically for dense-phase CO_2_ service. Recent work on UNS N06625 weld clads on carbon steel and on nickel–alloy UNS N06625 welds exposed to CO_2_/H_2_S at 120–200 °C and high H_2_S partial pressure indicates that Ni-based weld overlays and welds can provide robust resistance in sour supercritical conditions, but corrosion resistance depends on controlling dilution and microstructure to avoid iron-rich regions and localized attack [174,175,176].

Material selection is challenging due to potentially variable acid chemistry. For instance, H_2_SO_4_-dominated systems are very different from those systems where HNO_3_ may also form because HNO_3_ leads to extremely high corrosion rates and requires more resistant alloys [72]. Furthermore, due to transient conditions, liquids that condense at lower temperatures could subsequently warm locally and become extremely corrosive, so higher-temperature corrosion resistance must be considered. For all these reasons, material selection must consider the dropout mechanism, including temperature–pressure windows, impurity composition, and mixing behavior [63,74,177,178]. Although corrosion-resistant alloys can provide strong protection against CO_2_-induced corrosion, their potential use is limited by high alloy costs, associated fabrication complexity, challenging repairs, and compatibility issues with other materials in mixed-metal systems. To better select and qualify durable, low-impact alloys for large-scale deployment, future work should focus on more economical, fabrication-friendly alloy families and on advanced modeling/simulation tools that can predict corrosion response across relevant CCS conditions.

### 4.4. Protective Coatings

Protective coatings can be useful, particularly where localized acid dropout cannot be fully prevented, by acting as physical barriers between the metal surface and electrolyte, reducing susceptibility to pitting and under-deposit corrosion. Some coatings act as barriers, while others provide sacrificial shielding, limiting corrosion damage to the coating instead of the underlying metal surface. Organic coatings, polymer nanocomposites, and nickel–phosphorus (Ni-P) coatings are commonly used coatings.

Organic coatings, including fusion-bonded epoxy, epoxy–phenolic, and polyurethane, are commonly used as internal coatings in some CO_2_ systems to provide a barrier between steel and corrosive fluids and to improve hydraulic efficiency. However, the experience from the oil and gas industry indicates that conventional organic coatings can suffer from CO_2_-induced blistering, permeation, and mechanical damage, and defects can become focal points for severe under-film corrosion if acidified water penetrates [5,179]. To overcome these issues, more robust coating systems, including polymer nanocomposite coatings and thermal-spray or cold-spray metallic coatings, are required [180,181]. Studies highlight that clay- and graphene-reinforced polymer nanocomposite coatings, exhibiting reduced CO_2_ permeability and improved barrier performance compared with neat polymers, have the potential to enhance resistance to wet, impure CO_2_ and acidifying condensates [182,183].

Ni-P coatings show good corrosion resistance in a CO_2_ environment, but the co-presence of H_2_S leads to an increase in the diffusion at the interface between the coating and electrolyte, and the electrolyte can easily penetrate through the coating; hence, local corrosion and coating peeling occur [184]. The Ni-Cr-Mo coatings can be beneficial, as they have shown higher corrosion resistance in simulated solution environments containing CO_2_, H_2_S, and their mixture. Incorporation of other elements or compounds into Ni-P coatings can further improve the coating performance and their environmental stability [185,186]. Similarly, thermal-spray coatings of CRAs (for example, HVOF-based, Ni-based, and stainless steel alloys) have the potential to provide a promising solution, as they are sufficiently dense, well-adhered barrier coatings capable of withstanding service under supercritical, wet CO_2_ and H_2_S conditions. These coatings are being actively evaluated and marketed for CCS pipelines and vessels, although their performance remains sensitive to defects, coating microstructures, and test conditions [187,188,189]. S. Paul and his colleagues have extensively worked on thermal-spray coatings evaluated for supercritical/dense-phase CO_2_ and CCS environments (Table 9).

For acid dropout conditions, coatings have to endure not only chemical attack by H_2_SO_4_ and HNO_3_ but also mechanical and environmental stresses associated with temperature (including diurnal fluctuations) and pressure cycling (fatigue), low temperatures, and CO_2_ permeation. It is important to note that apart from a limited number of autoclave and flow-loop studies in model CO_2_/H_2_S brines, there is very little published information on the long-term performance of polymer, metallic, or ceramic coatings under realistic dense-phase CO_2_ conditions containing mixed SO_x_/NO_x_/O_2_/H_2_S and repeated temperature/pressure cycling. Hence, coating can be suitable for short, high-risk sections or as internal linings in controlled drop-out zones, rather than a stand-alone approach to mitigate acid dropout-related corrosion. Polymer-based coatings may suffer from plasticization, blistering, or chemical degradation in a highly acidic, CO_2_-rich environment, due to CO_2_ absorption during transport [195,196]. Long-term performance of organic coatings in dense/supercritical CO_2_ is important but poorly quantified for CCS, but the mechanisms (swelling, plasticization, property changes) in dense/supercritical CO_2_ are analogous to those in bulk polymers. Available data show that elastomer and thermoplastic polymer selection for CCS must consider not only chemical resistance properties but also CO_2_ sorption, swelling, and decompression behavior [197]. For example, fluorinated polymers and high-acrylonitrile HNBR can show significant volumetric swelling and severe effects under rapid gas decompression (RGD). Similarly, liquid CO_2_ can extract additives such as plasticizers from polymers, altering their mechanical properties. Crosslinked PE, due to its crystallinity and crosslinking, may limit CO_2_ sorption and swelling, showing promising performance for highly pressurized conditions. Coatings, including polymer, metallic, or ceramic coatings, must be resistant to acid attack and thermal cycling. Integrating coatings with CRA substrates or applying localized cladding in high-risk zones may provide a more robust solution. However, to ensure effectiveness, it is essential to evaluate coating performance under representative co-condensation and acid dropout conditions.

Table 10 summarizes coating types being considered for carbon capture systems and their potential role in mitigating corrosion under acid dropout conditions.

### 4.5. Simulation and Prediction Modeling

Simulation and predictive modeling are important tools for mitigating corrosion due to acid dropout, as they detect when and where corrosive acid-based liquid phases are likely to form and quantify the associated corrosion rates before actual damage occurs.

Thermodynamic and speciation models (EOS plus electrolyte frameworks) have been used to predict water and acid solubility, the onset of free water or acid-rich phases, and the composition/pH of any condensate for given mixtures of CO_2_, H_2_O, SO_2_, NO_2_, O_2_, H_2_S, and other impurities [64,205,206]. These models are usually coupled with multiphase flow and hydraulic simulations to identify where temperature–pressure profiles cross phase boundaries and acid-rich droplets may appear [66,96,207,208]. The application of these models includes defining safe impurity envelopes and operating windows where the probability of acid dropout is minimized, and identifying critical sections (low points, valves, decompression zones) where material upgrades or additional monitoring are necessary [90,96,209,210]. However, for CCS-related multi-impurity combinations and transient operating conditions, the validity of these models remains restricted by the limited laboratory data, in which important processes such as non-equilibrium acid nucleation, local mass-transfer limitations, and multi-impurity reaction kinetics are poorly controlled. Therefore, current simulations are more appropriately considered decision-support tools, complementing strict specifications, focused material improvement, or monitoring, rather than a replacement for practical data or safety factors.

To reflect the effects of strong acids and the interactions between impurities, traditional wet-CO_2_ corrosion models for oil and gas service, such as the de Waard–Milliams-type semi-empirical approaches used in wet gas and CO_2_-EOR systems, are being applied to situations where condensate composition and pH are known. Recently, electrochemical kinetics for sulfuric and nitric acid corrosion has been integrated with transport models to estimate local corrosion rates within condensate films and droplets formed from dense-phase CO_2_ with SO_2_/NO_2_/O_2_, capturing the observed transition from negligible corrosion to higher rates once acid-rich phases are formed [63,211,212].

To visualize the distribution of shear stress, mass-transfer coefficients, and local risk of corrosion at such complex geometries as tees, expansions, and low points, computational fluid dynamics (CFD) has been combined with corrosion models, which can demonstrate the influence of hydrodynamic focusing on the intensity of corrosion attack in the regions where acid droplets or films are present [213,214,215]. While numerical pit-growth models of corrosion within the CO_2_-containing medium with organic acids and low pH have been used to explore how local chemistry and diffusion/reaction balance control pit stability, this can serve as a guide for the use of this approach on H_2_SO_4_/HNO_3_-rich condensates [216].

Molecular dynamics and related atomistic simulations are also being used to investigate how impurities such as H_2_O, O_2_, SO_2_, NO, and NO_2_ alter the local molecular structure and chemistry of supercritical CO_2,_ such as the clustering of H_2_O and acid precursors, and its interactions with steel and passive films, providing an understanding of the nucleation of aqueous clusters and their adsorption behavior at the solid–fluid interface [131,217]. It is essential to combine such molecular-scale understanding with continuum-scale corrosion and transport models to understand synergistic interactions between impurities and to predict critical concentration/temperature thresholds for acid formation [206].

Recently, many studies have been conducted to validate these thermodynamic/speciation and corrosion models against laboratory data or project-based observations for dense-phase CO_2_ with impurities. Sonke et al. recently compared the thermodynamic predictions from an OLI-type mixed-solvent electrolyte model with experimental limits for acid dropout in dense-phase CO_2_ with mixed impurities at 20–100 bar and −25 to 25 °C for 20–50 days. They found that the thermodynamic model is conservative relative to experiments, which means the model-predicted unsafe points were below practical thresholds. This happened because the model included only thermodynamic solubility and speciation, while non-equilibrium effects such as reaction kinetics and nucleation barriers were not represented. On the other hand, Faraji et al. studied the equilibrium stability diagrams for iron corrosion products in a CO_2_ stream with H_2_O, SO_2_, NO_x_, and H_2_S, and found that the model correctly reproduced the dominant scales (FeCO_3_, Fe-oxides/hydroxides, sulfides, sulfates, etc.) observed on carbon steel in autoclave tests performed at 20–100 bar and −25 to 25 °C [56]. Also, the model even showcased the transition from protective FeCO_3_ layers in low-oxidizing conditions to porous oxides and hygroscopic sulfates/nitrates at higher O_2_/NO_2_ levels. In a separate study, Sonke et al. combined chemical-equilibrium calculations (CECs) with dense-phase CO_2_ tests to identify acceptable impurity limits for several CCS hub projects and found that model-derived measures, including equivalent sulfuric acid concentration and OLI-type mixed-solvent electrolyte frameworks, can predict the limits of acid-rich phase separation when combined with experiments [93]. However, there is limited understanding of transient impurity histories, local temperature–pressure paths, condensate compositions, and corrosion rates along operating CCS pipelines. Table 11 summarizes the strengths and limitations of various monitoring, simulation, and prediction tools for acid dropout mitigation. 

In all, as of now, these thermodynamic, CFD, and corrosion models can be considered as decision-support tools, complementing typical impurity specifications, material selections, and monitoring, rather than independent prediction tools for safe operations.

### 4.6. Operational Monitoring and Integrity Management

Operational monitoring and integrity management are very important for mitigating acid dropout corrosion because they translate impurity- and water-control strategies into verifiable, continuously controlled operational practices throughout the entire CCS lifetime. In order to identify acid dropout-driven damage and ensure timely treatment, in-line inspection, and leak detection, combined with risk-based decision-making frameworks, were included in an operational monitoring program. This program includes continuous monitoring of CO_2_ composition and of critical operating parameters (pressure, temperature, flow) at capture outlets, entry points, and other critical locations in the network. Online analyzers such as gas analyzers, hygrometers, and chromatographic systems at capture outlets and pipeline entry points verify compliance with CO_2_ quality specifications, while SCADA data of pressure, temperature, and flow are checked against phase-behavior envelopes to avoid operating in regions where condensation and acid-rich droplets are predicted.

In practice, impurity monitoring in dense-phase CO_2_ is challenging and can misrepresent conditions related to acid dropout. Morland et al. demonstrated that online analyzers report artificially low impurity levels while substantial acid formation has already taken place due to pressure reduction and phase change in sampling systems, which can cause H_2_O, H_2_S, SO_2_, and NO_2_ to react and partition into acid-rich liquid phases and elemental sulfur [64,89]. From long-term autoclave experiments at 100 bar and 25 °C, it was found that even low-ppm mixtures of H_2_O, H_2_S, SO_2_, NO_2_, and O_2_ readily react, consuming 70% or more of the injected impurities in an acid-rich aqueous phase, with elemental sulfur formed, before reaching the analyzer. In one example, simultaneous injection of 300 ppm(v) H_2_O, 350 ppm(v) O_2_, and 100 ppm(v) each of NO_2_, SO_2,_ and H_2_S led to the formation of strong acid and sulfur. The online analyzers reported virtually no detectable H_2_S and a strongly reduced SO_2_/O_2_ ratio, apparently in-spec despite substantial acid production in the system. Therefore, CCS operators must design sampling systems carefully to maintain gas sample conditions (e.g., heated sample probes, short sample lines, provision for capturing liquids/solids) and interpret measurements in combination with thermodynamic and reaction modeling, rather than solely relying on a single parameter value as an indicator of safe operation [89,218].

Targeted corrosion monitoring of specific high-risk sites, for example, low-points, valves, and dead legs, using corrosion coupons and electrical-resistance (ER) probes, supported with (if suitable) electrochemical techniques (for example, LPR or EIS when a sufficiently conductive aqueous phase is present) and other non-destructive testing (NDT) methods, provides the degree of corrosivity and detects transient fluctuations in corrosion rate with specific impurity excursions or cooling events. For electrochemical probes to function reliably, a three-electrode configuration and a continuous conductive liquid phase are necessary, which limits their use to regions where aqueous films are expected. Emerging integrated monitoring systems couple impurity time series together with calculated condensate pH and in situ corrosion data into a single dashboard, enabling operators to recognize characteristic signatures of acid dropout events, such as simultaneous increases in NO_2_/SO_2_, a decrease in local temperature, and increased corrosion rate at a specific station [90,218,219,220]. In some experiments, a sudden decrease in measured H_2_S after NO_2_ addition has been reported, likely showing its consumption in acid-forming or secondary reactions rather than simple removal from the system [95]. This kind of response should be interpreted together with speciation and corrosion data rather than in isolation.

Existing pipeline integrity management strategies can be adapted to and modified for CCS, incorporating acid dropout risk, by integrating scheduled in-line inspection (ILI), NDT, and cleaning with structured risk assessments and fitness-for-service evaluations [220]. Smart pig devices based on magnetic flux leakage and ultrasonic technologies can provide detailed pipeline wall-thickness and local metal-loss data. These may then be used to characterize internal corrosion features and locate localized corrosion damage sites, such as low points where acidic liquids may accumulate.

At first, cleaning and drying are performed to remove debris and water from the CCS network (repaired or new pipelines), then initial ILI runs are conducted to characterize the pre-existing corrosion and set reference conditions for subsequent monitoring. Thereafter, integrity strategies define inspection intervals, repair criteria, and the sites where upgrade options (e.g., coatings, CRA liners) must be considered for regularly wet or modeled high acid dropout sections, ensuring that mitigation measures address risks through both monitoring and predictive analysis [90,205,220].

Leak detection and emergency response procedures need to complement the integrity management strategy by incorporating computational leak-detection systems (pressure/flow balance, negative pressure waves) and a range of sensing technologies (fiber-optic, acoustic, or external chemical sensing) to detect even small leaks and locate them rapidly. By linking leak-detection alarms with impurity records, corrosion, and ILI data, operators can not only respond to incidents but also analyze the acid dropout mechanisms that led to damage, thereby refining specifications, operating envelopes, and inspection plans to prevent recurrence.

## 5. Conclusions, Challenges, and Future Directions

### 5.1. Conclusions

This review addresses the current understanding of acid formation and dropout in carbon capture systems, which are among the most critical corrosion threats to carbon-steel infrastructure. Dry CO_2_ (gas or dense phase) is essentially non-corrosive to carbon steel, but once an aqueous or acid-rich liquid phase forms through water condensation or acid dropout, there can be a highly localized corrosive environment, resulting in corrosion rates too high for long-term safe operation [65,143,149]. Experimental work has shown that sulfur- and nitrogen-containing impurities (SO_2_ and NO_2_), in the presence of trace H_2_O, can generate strongly acidic condensates dominated by H_2_SO_4_ and HNO_3_. The aqueous solutions obtained after collecting and diluting these condensates typically show pH values near or below two, leading to general corrosion rates of several mm·y^−1^ and localized penetration depth of hundreds of micrometers within days [72].

A key conclusion is that acid dropout corrosion severity appears to be controlled by the combined effects of individual impurities (H_2_O, SO_2_, NO_2_, O_2_, H_2_S, amines, glycols), their reaction pathways, phase partitioning between dense-phase CO_2_ and acidic phases, and local acid–base equilibrium, rather than by single-impurity limit. In dense-phase CO_2_ with mixed impurities, initially small acid-rich phases can become even more acidic and corrosive through the absorption of additional H_2_O and polar species, which could not have been detected from gas-phase specifications and bulk equilibrium calculations alone [64]. Therefore, acid dropout corrosion, which is controlled by thermodynamics (phase and speciation), reaction kinetics, mass transport, and operational transients such as depressurization, start-up, shutdown, and stream mixing, is best seen as a system-level phenomenon [65,90]. Consequently, mitigation must include integrated strategies that combine deep dehydration and strict impurity limits, segment-specific material selection (CRA and coatings in the targeted dropout zones), and operational controls that avoid or reduce the thermodynamic or kinetic probability of acid dropout [63,205].

In practice, dehydration combined with conservative impurity limits and definitive operating envelopes is the primary engineering strategy and currently used in CCS. Whereas other measures discussed in this review, such as chemical inhibition, scavengers, advanced coatings, and the use of some CRAs in selected segments, are either validated mainly by short-term lab-based data or are derived from oil and gas scenarios, and their performance in dense-phase CO_2_ under the actual multi-impurity profile and transient conditions remains unclear. On the basis of currently available evidence, from an engineering design viewpoint, dehydration and impurity control can be considered the first line of defense, while CRAs and coatings should be used in targeted dropout zones within carefully defined applicability envelopes and only after further qualification under real mixed-acid conditions.

### 5.2. Key Challenges

**Figure 5:**
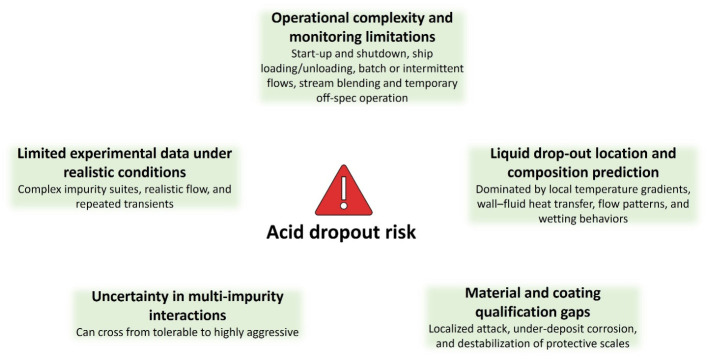
Key challenges associated with acid dropout risk in carbon capture transport systems.

Even though substantial progress has been made, the evidence summarized in this review also highlights that several technical and knowledge gaps continue to limit effective corrosion-risk management for CO_2_ transport and storage (Figure 5).

- Limited experimental data under realistic conditions: The most detailed quantitative data currently available for mixed-acid systems comes from static or semi-static autoclave tests, involving simplified chemistries. The data show that the vast majority comes from traditional oil and gas studies conducted at relatively moderate pressures and within the context of CO_2_-HO_2_ systems. CCS systems involve dense-phase CO_2_, with complex impurity profiles, realistic flow, and repeated transients. In the past decade, CCS-focused projects in Norway and other places have generated valuable high-pressure data for dense-phase CO_2_ with controlled SO_2_/NO_2_/O_2_/H_2_O/H_2_S mixtures, but there have been limited numbers of systematic dynamic studies that combine dense-phase CO_2_, controlled impurity mixtures, continuous electrochemical monitoring, and representative hydrodynamics. Detailed data is essential for defining parameters and validating mechanistic models for acid dropout corrosion [90].
- Uncertainty in multi-impurity interactions: In practice, CO_2_ streams would generally contain multiple impurities simultaneously; however, many specifications and models still treat each impurity independently. Experiments have shown remarkable synergies among NO_2_, SO_2_, O_2_, and trace water (and sometimes H_2_S), where individually safe mixtures become a source of highly acidic condensates and severe corrosion when a liquid phase forms [72,206]. It remains a significant challenge to predict when an actual impurity blend would shift from tolerable to highly aggressive behavior, particularly during transient conditions.
- Prediction of liquid dropout location and composition: Thermodynamic tools might be useful in describing bulk-phase equilibrium; however, predicting when and where and with what composition is very difficult because of local temperature gradients, heat transfer at the wall–fluid interface, flow patterns, and wetting behaviors that influence the formation of corrosion products as well as acidic droplets [217]. In addition, the kinetics governing the nucleation, growth, and removal of these corrosion products are difficult to predict in complex geometries and under transient flow conditions. The local solubility limit of water or acid in CO_2_ can be exceeded during start-ups, shutdowns, pressure drops, or the mixing of streams with different temperatures or impurities, but current models and monitoring methods cannot resolve these effects due to transients [214]. Recently, Sonke et al. have compared the equilibrium solubilities of H_2_SO_4_ using autoclave tests [93]. They found that acid precipitation occurred only when the acid concentration was around 20–30 times higher than the thermodynamic solubility. This could be due to slow chemical conversion and nucleation kinetics, indicating that both thermodynamics and kinetics must be considered when assessing the potential for dropout and corrosion in CCS.
- Material and coating qualification gaps: Carbon steel can be suitable when CO_2_ is appropriately dried, and impurities are tightly controlled, but environments where acid dropout is possible require CRAs or internal linings/coatings for increased lifetimes. Since there is limited data available for CRA, CRA clad systems, and advanced coatings performance in CO_2_-rich mixed-acid dropout conditions, especially for localized attack, under-deposit corrosion, and destabilization of protective scales, the current selection parameters include a high degree of uncertainty [5,133,150,205]. Although NACE (now AMPP) used to have guidelines for acid service, they were based on single-acid environments such as H_2_SO_4_ or HNO_3_. Some CRAs can be more resistant to HNO_3_ than H_2_SO_4_ and vice versa. In CCS, where mixed-acid dropout conditions with constantly changing relative ratios are very common, it is highly uncertain whether alloys validated for single acids can be used in mixed-acid environments. Hence, these CRAs and coatings need to be studied in targeted simulated environments.
- Operational complexity and monitoring limitations: Real operating scenarios, such as start-up and shutdown, ship loading/unloading, batch or intermittent flows, stream blending, and temporary off-spec operation, are often under-represented in design specifications, even though they can drive systems temporarily outside nominal operational envelopes and greatly increase the likelihood of condensation and acid formation. For instance, in the case of shipborne CO_2_, it could be challenging if small amounts of acid form and dropout in the cargo tanks during unloading. Subsequent loading and unloading cycles might further accumulate acids in lower regions, whereas standard bulk-phase sampling may fail to detect their presence, leaving the tank’s actual internal conditions uncertain. Strategies such as controlled dropout zones, scavenger addition, or tight impurity control demand high levels of operational discipline, robust impurity and corrosion monitoring, and rapid response, which are challenging to implement consistently across large, multi-user hub-type CCS networks [218,220]. Furthermore, as Morland et al. showed, the sampling and analysis of dense-phase CO_2_ streams can themselves be biased by in-line reactions and phase changes [89]. Hence, to detect localized acid accumulation in tanks and pipelines, there is a need for improved diagnostic methods, preferably non-invasive or minimally intrusive.

### 5.3. Future Research Directions: Experiments, Modeling, Standards, and Materials

To address the aforementioned challenges and enable safe, large-scale CO_2_ transport and storage, coordinated advances in experimental, modeling, specification, and mitigation technologies are required.

- Experiments: It is important that future studies focus on dynamic corrosion experiments in dense-phase CO_2_ with well-controlled impurity compositions, realistic flow conditions, and temperature/pressure fluctuations, combined with continuous monitoring and detailed post-exposure characterization of corrosion products. In practice, conventional electrochemical techniques such as LPR and EIS are difficult to apply directly to dense, non-conductive CO_2_, especially when only thin water films or small droplets are present on the metal surface. Therefore, it is necessary to adapt or develop monitoring techniques compatible with such environments and to combine them with high-resolution post-test methods (such as 3D profilometry) to quantify highly localized metal loss at acid dropout sites. Likewise, it is important to develop and standardize test methods that minimize experimental artifacts such as incorrect orientation and placement of specimens, applying EIS, LPR techniques in thin water films, poor electrode configuration, or unintentional water condensation on the test specimens during depressurization [222,223].
- Modeling: There is a clear need for the development of next-generation prediction tools that combine phase-equilibrium calculations with multi-impurity reaction kinetics, mass transport, and electrochemical corrosion models to enable location-specific predictions of condensate composition and corrosion rate under transient conditions. Such models should focus on (i) tracking the evolution of condensate composition on steel surfaces; (ii) calibrating and validating against detailed laboratory data from mechanism-based experiments; and (iii) incorporating developed models into digital twin frameworks combining process simulation, corrosion prediction, monitoring, and inspection data to support proactive risk assessment and optimized maintenance.
- Standards and specifications: Since single, conventional limits for impurities would not be appropriate, CO_2_ quality specifications in the future should be risk-based and context-dependent, considering impurity combinations, transport mode (pipeline, ship, truck), construction materials, and expected transient operations. Research work in this area should focus on (i) developing corrosion-driven impurity envelopes from the new experimental and modeling work; (ii) defining measurable indicators like maximum acceptable condensed-phase acidity; and (iii) implementing these indicators into further revisions of CO_2_ quality standards and recommendations for CCS pipelines, ships, and wells.
- Materials: For materials, future research needs to focus on establishing the applicability envelopes of corrosion-resistant alloys, CRA claddings (weld, laser, etc.), and advanced functional coatings (e.g., nanocomposite or spray systems) in mixed-acid scenarios, mimicking the realistic impurity profiles rather than single-acid solutions. Research tasks should also include (i) the mapping of localized corrosion and cracking thresholds for different CRAs in dense CO_2_ with typical SO_x_/NO_x_/O_2_/H_2_S levels; (ii) the assessment of welds and weld overlays; and (iii) the evaluation of hybrid protection concepts, combining CRA liners with robust internal coatings (e.g., nanocomposite or spray systems) on a carbon steel base within the identified dropout zones and other high-risk regions.

### 5.4. Outlook

This review summarizes the understanding based on a wide range of experimental, modeling, and field data, but several limitations should be focused on. Most of the mixed-acid datasets are obtained from short-term autoclave tests with simplified chemistries, but long-duration, dynamic experiments in dense-phase CO_2_ with actual multi-impurity blends, transients, and hydrodynamic conditions are still unclear. Also, performance data for CRAs, welds, clads, and coatings under mixed-acid dropout conditions is limited. From the discussed mitigation strategies and mechanistic speculations, some are proven while others should be treated as working hypotheses or rational deductions rather than fully proven solutions, such as the kinetics of acid nucleation and growth in dense CO_2_, proposed acid dropout impurity threshold under transient flow, safe operating envelopes of individual CRAs in highly oxidizing and highly saline condensates that are still limited or estimated from the related environments. The future research directions outlined in Section 5.3 are proposed to convert these rational conclusions into validated design tools so that they can support the progression of standards from common impurity limits towards context-specific profiles. Such developments would help distinguish between mitigation options that are proven effective and those that are promising yet still developing, ultimately facilitating the large-scale deployment of CCS systems as part of global decarbonization strategies.

## Figures and Tables

**Table 1:** Direct carbon capture plants in development. Reproduced from [23] under CC BY 4.0 license.

Project/Plant Name	Carbon Capture Capacity (tCO_2_/Year)	CO_2_ Use/Storage	Target Operation Date	Country
DAC pilot plant	365	Storage (injection)	2022	Australia
Haru Oni eFuels pilot plant	-	Use (synthetic fuels)	2022	Chile
Norsk e-fuel project	-	Use (synthetic fuels)	2023	Norway
DAC 1 project	1 million	Storage (injection)	2025	USA
Dreamcatcher project	Up to 1 million	Storage (injection)	2026	UK
Air-to-fuels plant	-	Use (synthetic fuels)	2026	Canada
Atoms FUEL project	-	Use (synthetic fuels)	2029	UK
Sizewell C nuclear-powered DAC	100	Storage (injection)	-	UK
Kollsnes project	Up to 1 million	Storage (injection)	-	Norway

**Table 2:** Summary of typical impurity profiles from different carbon capture processes and factors affecting these impurity compositions.

Capture Process	Main Impurity Profile	Factors Affecting the Impurity Profile	Remarks
Post-combustion process	CO_2_ with trace O_2_, N_2_, H_2_O, solvent residues, and residual SO_x_/NO_x_	Flue gas treatment efficiency (FGD, ESP, SCR) Solvent type and degradation Furnace conditions Dehydration and compression efficiency	Remaining water and acid gases can cause corrosion; solvent residues may introduce new reactive species
Pre-combustion process	CO_2_ separated from syngas; may contain H_2_, CO, H_2_O, H_2_S, COS, CO	Gasifier type Fuel composition (sulfur content, ash level) Solvent type Gas-cleanup and dehydration method	Sulfur-containing species can react with O_2_ and NO_x_ when mixed with oxidizing streams
Oxy-fuel combustion process	High-purity CO_2_ with residual O_2_, N_2_, Ar, H_2_O, SO_x_ and NO_x_	ASU oxygen purity Fuel composition Air ingress FGD/SCR efficiency CO_2_ purification method Operation conditions	After purification, the remaining H_2_O and acid gases can increase the risk of acid formation
Direct air capture	Very low sulfur and nitrogen; mainly H_2_O, O_2_, sorbent/solvent residues	Sorbent/ solvent type Ambient humidity Process configuration Dehydration and compression efficiency	Moisture and process-related contamination can cause dehydration and downstream mixing
CCS hub (mixed carbon capture processes)	Dense-phase CO_2_ with a wide range of impurities from various emitters	Stream mixing ratio of oxidizing and reducing streams Kinetics and thermodynamics of the impurity mixture Temperature and pressure fluctuations Flow conditions and residence time	Very uncertain impurity behavior Acid dropout and localized corrosion may occur due to impurity interactions and phase separation

**Table 3:** Typical CO_2_ concentrations in flue gases from various sources. Refs. [31,32,33,34]. Reproduced from [31] under the Creative Commons CC BY 4.0 license.

Flue Gas Source	CO_2_ Conc. (vol. %)	P (atm)
Gas turbine	3–4	1
Fired boiler of oil refinery and petrochemical plant	~8	1
Natural gas-fired boilers	7–10	1
Oil-fired boilers	11–13	1
Coal-fired boilers	12–141	1
IGCC after combustion	2–14	1
Hydrogen production	15–20	22–27
Steel production (blast furnace)	20–27	1–3
Aluminum production	1–2	1
Cement process	14–33	1

**Table 5:** Comparison of experimental CCS acid dropout and impurity-corrosion studies under equivalent conditions.

CO_2_ Phase	Material	T (°C)	P (bar)	Impurities Injection	H_2_O (ppm(v))	O_2_ (ppm(v))	SO_2_ (ppm(v))	NO_2_ (ppm(v))	H_2_S (ppm(v))	Corrosion Rate	Remarks	Ref.
Dense/Saturated	Carbon steel	4–40	95	-	0	0	0	0	0	<2 µm/y; FeCO_3_ products observed	Useful baseline, but not an acid dropout case.	[83]
Dense	Carbon steel	25	100	Simultaneous	300	350	100	100	100	0.2 mm/y	Large amount of sulfur formed; H_2_SO_4_:HNO_3_ ≈ 20:1	[84]
Dense	Carbon steel	45	100	Simultaneous	300	350	100	100	100	0.05 mm/y	Low corrosivity at higher temperature; H_2_SO_4_:HNO_3_ ≈ 35:1	
Dense	Carbon steel	25	100	In series	300	350	100	100	100	0.04 mm/y	Acid dropout; lower sulfur than simultaneous injection	
Dense	Carbon steel	45	100	In series	300	350	100	100	100	0.1 mm/y	Acid dropout with sulfur formation	
Dense	Carbon steel	25	100	Simultaneous	122	275	69	96	130	0.04 mm/y	Acid dropout (H_2_SO_4_:HNO_3_ ≈ 16:1) with sulfur formation	[85]
Dense	Carbon steel	25	99	In series	90	70	30	32	36	0.1 mm/y	Acid dropout (H_2_SO_4_:HNO_3_ ≈ 10:1) with sulfur formation	[86]
Dense	Carbon steel	25	99	In series	100	12	5	5	6	-	Below acid dropout threshold; no liquid acid phase; full conversion of H_2_S and O_2_	[87]
Dense	Carbon steel	25	99	In series	35	31	12	10	10	-	Near-threshold case; hint of solids but no liquid acid	
Dense	Carbon steel	25	99	In series	120	95	38	26	41	-	Acid dropout; liquid acid phase; small amount of sulfur	

**Table 6:** Combinations of impurities and their reaction outcomes.

Impurity Combination	Key Reactions/Products	Dominant Acid/Phase Formed	Risk Level	Notes/Mechanistic Insights
H_2_O + SO_2_	Dissolution of SO_2_ → H_2_SO_3_	Weak acid, mostly dissolved	Low	Slow kinetics; limited drop-out unless water is abundant
H_2_O + SO_2_ + NO_2_	NO_2_ oxidizes SO_2_ → SO_3_ → H_2_SO_4_	H_2_SO_4_ (strong acid)	Very high	NO_2_ acts as a strong initiator; drop-out is observed even at low-ppm levels
H_2_O + NO_2_	Hydration/oxidation → HNO_2_ (intermediate) → HNO_3_	HNO_3_ (strong acid)	High	HNO_3_ has high solubility, but is extremely corrosive when condensed
H_2_O + SO_2_ + O_2_	O_2_ oxidizes SO_2_ → SO_3_ → H_2_SO_4_	H_2_SO_4_	Moderate–high	Requires higher-impurity concentrations; kinetics slower than NO_2_-driven reactions
H_2_O + H_2_S + SO_2_ + O_2_	Redox reactions → elemental Sulfur	S^0^ solid	Moderate	Competing pathways: Sulfur vs. sulfuric acid, depending on the abundance of O_2_
H_2_O + H_2_S + NO_2_ + SO_2_	H_2_S + NO_2_ → SO_2_ + NO → H_2_SO_4_ formation	H_2_SO_4_ (low threshold)	Very high	Worst-case combination; NO_2_-H_2_S radical chain accelerates the reaction drastically
H_2_O + H_2_S + NO_2_ + O_2_ + SO_2_	Multiple cycles: NO ↔ NO_2_, SO_2_ oxidation, H_2_S oxidation	H_2_SO_4_ and/or HNO_3_	Extreme	Automatic regeneration of NO_2_; complex network leads to rapid acid formation
H_2_O + NO_2_ + O_2_ + SO_2_ (no H_2_S)	NO_2_-SO_2_ route dominates	H_2_SO_4_	High	Fewer pathways but strong acid formation due to efficient SO_2_ oxidation

Note: Oxidation of H_2_S in the presence of O_2_ and NO_2_ can yield overall stoichiometries in which H_2_O appears as a product (for example, H_2_S + 1/2O_2_ → S + H_2_O; H_2_S + 3/2O_2_ → SO_2_ + H_2_O, and multi-component S-N-O-H balances) [93]. However, in dense-phase CO_2_ context, H_2_O is primarily introduced as an impurity, and the formation of strong acids (H_2_SO_4_ and HNO_x_) and sulfur-containing solids is the main concern of these reactions.

**Table 7:** Examples of corrosion behavior of pipeline steel in the presence of various acid-forming impurities in dense CO_2_.

Material	Conditions (P, T, Duration)	SO_2_	NO_2_	Other Species (H_2_S, O_2_, H_2_O)	Corrosion Rate (mm/Year)	Corrosion Products	Reference
Carbon steel	80 bar, 50 °C, CO_2_-saturated water and water-saturated dense CO_2_	0 and 0.8 bar	-	O_2_ varied (0–0.4 bar) in CO_2_-saturated water and water-saturated CO_2_; H_2_O as bulk aqueous phase or water-saturated CO_2_; no H_2_S	In CO_2_-saturated water without impurities, high general corrosion; in water-saturated CO_2_ without impurities ~0.38 mm/y; addition of 0.8 bar SO_2_ increased rate to 5.6 mm/y; with both O_2_ and SO_2_ > 7 mm/y	Without SO_2_, mainly FeCO_3_ films (partially protective); with SO_2_, FeSO_3_·3H_2_O forms, then oxidizes to FeSO_4_ and FeOOH in the presence of O_2_, giving porous, non-protective scales	[143]
X70	Supercritical CO_2_ at 10 MPa, 50 °C; exposure times typically up to 7 days	varied from 0 to several hundred ppm	-	O_2_ varied (up to several hundred ppm) in sCO_2_; H_2_O at saturation with sCO_2_; no H_2_S	O_2_ alone had negligible influence on corrosion rate in sCO_2_; increasing SO_2_ increased corrosion rate; O_2_ + SO_2_ produced the highest rates (exact values depend on SO_2_ level)	In pure CO_2_/H_2_O, FeCO_3_-rich films; with SO_2_ and especially SO_2_ + O_2_, films become more porous and enriched in sulfur-containing species	[144]
L360NB	CO_2_ gas mixtures containing controlled H_2_O, O_2_, NO_2_ and SO_2_; condensed acid solutions at pH ≈ 2.13 with varying HNO_3_/H_2_SO_4_ ratios; exposure over hours–days	SO_2_ present in CO_2_ mixtures, producing H_2_SO_4_ in condensate; concentrations varied to change H_2_SO_4_ fraction at fixed initial pH	NO_2_ present, generating HNO_3_; NO_2_/SO_2_ ratio adjusted, changing HNO_3_/H_2_SO_4_ ratio at fixed initial pH	H_2_O as thin condensate films; O_2_ present in CO_2_ mixtures; no H_2_S	Overall rates for carbon steel in these pH ≈ 2 acids were of the order 1–2 mm/y, with significant pitting in mixed-acid, chloride-bearing cases	In HNO_3_-rich solutions, nitrate-dominated films are not protective, favoring active dissolution; in H_2_SO_4_-rich solutions, sulfate-containing scales form but remain insufficiently protective; pit morphology and severity depend on the HNO_3_/H_2_SO_4_ ratio and chloride content	[72]
Pipeline steel	sCO_2_ (9.5 MPa, 60 °C, 1512 h)	SO_2_ added at increasing concentration (0–5%)	-	H_2_O: 488 and 1220 ppm(v); no O_2_	General corrosion rate increased ~2× with increased SO_2_ concentration (0.0109 to 1.396); corrosion rate slowed with exposure time as the reaction consumed a fraction of SO_2_	iron carbonate and sulfur-containing products (e.g., FeCO_3_ mixed with sulfite/sulfate phases) whose protectiveness decreased with increasing SO_2_	[145]
X52 and GR70 carbon steels and 9Cr alloy	Dense CO_2_ (100 bar, 5 °C and 25 °C),	SO_2_ present at low levels (60 ppm)	NO_2_ (0 or 100 ppm)	H_2_O (70, 350, and 700 ppm(v)), O_2_ (200 ppm), no H_2_S	With 70 ppm(v) H_2_O+ no NO_2_, low-moderate corrosion; with NO_2_, corrosion rate increased (for X52 and GR70 corrosion rate was 0.065 mm/y and 0.016 mm/y higher (~5× and 3× higher) at 5 °C vs. 25 °C; low H_2_O + NO_2_ at 5 °C gave rates up to 5× higher than at 25 °C	Primary corrosion product was FeSO_3_; with 100 ppm NO_2_, the corrosion product was FeO(OH) at <350 ppm(v) H_2_O; at 700 ppm(v) H_2_O FeCO_3_ and FeSO_3_ were the dominant corrosion products; 9Cr alloy more resistant than X52/GR70	[80]

**Table 8:** Different CRA types considered for CCS applications [150,157,162,163,164,165,166,167,168,169].

CRA Type	Uses in Carbon Capture Systems	Advantages	Limitations
13Cr martensitic stainless (standard 13Cr)	Older CO_2_ injection wells (e.g., Sleipner casing joints), some tubing/flowlines in sweet or mildly sour wet CO_2_	Relatively low cost compared to duplex and Ni-alloys; Better than C steel in simple wet CO_2_; Well-established for oil and gas industries	Not suitable where SO_2_, NO_X,_ and O_2_ can generate strong acids
Super 13Cr martensitic stainless (S13Cr)	CO_2_ injection tubing and well components in wetter, higher salinity, mildly sour conditions	Better performance and higher pitting resistance than 13Cr; Attractive where H_2_S and brine are present, but SO_2_/NO_X_ are low	Passive film is removed by strongly acidifying impurities; Shows unacceptable corrosion in SC-CO_2_ with SO_2_ + O_2_, so it cannot be considered robust against acid dropout driven by SO_2_/NO_2_
22Cr duplex stainless (e.g., UNS S31803/S32205)	Topsides, wellheads, and some pipeline/riser sections in CO_2_ injection projects	Good balance of corrosion resistance, strength, and cost; Widely used in existing CO_2_ injection projects; More tolerant to off-spec wetting than C steel or 13Cr	Pitting/crevice susceptibility increases in very low pH, high chloride, oxidizing acid condensates; Careful assessment needed when significant SO_2_/NO_X_ are possible
25Cr super-duplex stainless (e.g., UNS S32750/S32760)	CO_2_ injection tubulars in severe wet/sour service; high-risk CCS zones (near-wellbore, topsides where wet CO_2_ and impurities are expected)	High corrosion resistance to both CO_2_ and H_2_S; Already been used in CO_2_ injection; Strong candidate where acid dropout cannot be ruled out	More expensive and mechanically less strong than C steel; Needs careful welding and fabrication; Risk of hydrogen-assisted cracking and crevice corrosion in extremely aggressive, low pH, high-chloride environments; Still needs evaluation for specific carbon capture uses
Ni-based alloys (e.g., Alloy 625, C-276)	Cladding/overlays on C-steel, CRA-lined sections, critical valves, and well components where worst-case acid dropout is expected, and impurity control is uncertain	Outstanding resistance to acidified CO_2_ environments; Can be used as a weld overlay or clad pipe to protect high-risk zones; Resilient to variation in impurity levels and pH	Very high material and fabrication cost, so not suitable for long distances (suitable for short, high-risk zones); widely used in oil and gas, but thermal expansion mismatch and weldability issues when overlaid on C-steel
Modified low-Cr/Cr-containing pipeline steels (developmental CCS grades)	Potential CRA candidates for supercritical CO_2_ transport, small additions of alloying elements can improve corrosion resistance compared to conventional C steel.	Cheaper than other CRAs; suitable for long pipelines; May provide resistance against minor acid dropout in combination with tight impurity control	Performance envelope for strong acid dropout (H_2_SO_4_/HNO_3_) not yet fully established

**Table 9:** Examples of thermal-spray coatings evaluated for supercritical/dense-phase CO_2_ and CCS environments.

Coating Systems	Thermal Spray Methods	Substrate	Test Conditions	Observations	References
CRA coatings of UNS R50250, UNS N10276, UNS N06625 and UNS S31603	HVOF	carbon Steel	3.5 wt.% NaCl solution with 9.5 MPa CO_2_ and 0.5 MPa H_2_S at 40 °C for 30 days	Dense, well-adhered coatings with no scale; CRA layer protects steel in CO_2_/H_2_S; localized attack controlled by porosity and defects	[181]
CRA coatings of UNS R50250, UNS N10276, UNS N06625 and UNS S31603	HVOF	carbon steel	3.5 wt.% NaCl solution with 10 MPa CO_2_ at 40 °C and 80 °C for 30 days	Coatings remained dense and adherent with no visible scaling; Temperature influences performance; Coating defects affect local corrosion at the steel interface	[190]
CRA coatings of UNS N10276, UNS N06625 and UNS S31603	HVOF	carbon steel	3.5 wt.% NaCl solution with 50 MPa CO_2_ at 40 °C for 30 days	High-pressure supercritical CO_2_ exposure did not produce scaling on intact CRA coatings; Coating porosity and splat structure remain critical to long-term barrier performance	[191]
CRA coatings of UNS N10276, UNS N06625 and UNS S31603	HVOF	carbon steel	3.5 wt.% NaCl solution with 10 MPa CO_2_ at 40 °C and 80 °C, and 50 MPa CO_2_ for 30 days.	Intact CRA coatings provided effective protection over a range of T and P; In specimens with holidays, galvanic coupling and interfacial corrosion were present, highlighting the need to minimize through-porosity and seal defects	[192]
Thermally sprayed aluminum (TSA)	Twin-wire arc spray (TWAS) with aluminum	Carbon steel	3.5 wt.% NaCl solution with 10 MPa CO_2_ at 40 °C for 168 h	TSA coatings remained intact and well-adhered; Sacrificial Al layer limited general corrosion of the substrate in dense/supercritical CO_2_ brine	[193]
TSA-coated steel with a 5% holiday/defect	Twin-wire arc spray (TWAS) with aluminum	Carbon steel	3.5 wt.% NaCl solution with 10 MPa CO_2_ at 40 °C for 168 h	No corrosion observed at the TSA–steel interface and no accelerated corrosion near the exposed steel; Early results are promising but long-term CCS performance and larger defect sizes still need evaluation	[194]

**Table 10:** Summarized potential coating systems for acid dropout corrosion mitigation in carbon capture systems.

Coating Type	Typical Examples	Strengths	Key Limitations	References
Conventional organic coatings	Fusion-bonded epoxy (FBE), epoxy–phenolic linings, modified epoxies	Widely used and cost-effective; Easy application over long pipelines; Good barrier protection Good chemical resistance in many brine/CO_2_ environments; Improves flow efficiency	Susceptible to CO_2_ aging, permeation and blistering, Risk of debonding under high-pressure CO_2_; Coating defects may lead to severe under-film corrosion	[198,199]
Polymer nanocomposite coatings	Epoxy matrices with layered silicate clays or graphene nano-fillers	Better barrier properties Enhanced durability than neat polymers; Better resistance to CO_2_-induced plasticization and to delay acid penetration	Still under development for CCS pipelines; Dispersion quality and filler content are critical; Long-term behavior under acidic condensates is not fully understood	[179,200]
Electroless Ni-P coatings	High-phosphorus Ni-P deposits on carbon steel.	Good wear resistance and corrosion resistance; Relatively uniform deposition on complex geometries; Useful for localized protection (e.g., valves, fittings)	Performance degrades in strongly acidic mixed CO_2_/H_2_S environments and likely under highly acidic (H_2_SO_4_/HNO_3_-rich) dropout; Risk of localized corrosion if damaged	[201,202]
Ni-Cr-Mo laser/thermal-spray coatings	Ni-Cr-Mo alloy layers produced by laser cladding or thermal spray on carbon steel	Provide a dense, well-adhered metallic barrier with excellent resistance to CO_2_/H_2_S attack; Good tolerance to acidic condensates; Suitable for local linings in high-risk zones (e.g., controlled dropout sections, wellheads).	Higher application cost Requires strict process control (porosity, dilution) are critical; Defects or incomplete coverage can lead to corrosion; Limited long-term data on dense-phase CO_2_ with multi-impurity acid dropout	[184]
Thermally sprayed aluminum (TSA) coatings	Twin-wire arc-sprayed Al on carbon steel	Provides sacrificial cathodic protection and barrier effect Well-adhered and dense coatings Good corrosion protection in dense or supercritical CO_2_	Limited long-term performance in H_2_SO_4_/HNO_3_ dropout and repeated temperature/pressure cycling Effectiveness varies with coating defects and porosity	[193]
Inorganic or hybrid ceramic coatings	Iron-phosphate-based, ceramic-reinforced organosilicon epoxies	Very low permeability Good chemical resistance; Suitable for particularly aggressive zones.	Limited industrial experience for CO_2_ pipelines, Repair and inspection challenges Limited data for H_2_SO_4_/HNO_3_-rich dropout.	[198,203,204]

**Table 11:** Monitoring, simulation, and prediction tools for acid dropout mitigation. [50,63,64,90,96,206,211,214,215,218,219,220,221].

Tool/Approach	Purpose	Strengths	Limitations
Thermodynamic models (EOS + MSE)	Phase behavior, dew-points, water/acid solubility, condensate composition	Define safe operating windows Support impurity specifications; Useful for sensitivity studies.	Equilibrium-based; Less reliable at predicting trace-level impurities, non-ideal mixing, and non-equilibrium nucleation in flowing systems.
Integrated thermo-kinetic-corrosion models	Coupled phase behavior, reaction kinetics, and corrosion rates	Can estimate localized corrosion risk under transient conditions; Link impurity envelopes to corrosion risk.	Still under development; Needs extensive real multi-impurity data for calibration and validation
CFD and multiphase flow simulation	Flow regime, shear, liquid holdup, stratification, droplet transport, and cold-trap locations	Identify high-risk locations Support targeted monitoring	High computational cost; Sensitive to model assumptions; Limited chemistry prediction
Digital twins (process + corrosion + integrity)	Real-time prediction of pipeline conditions and degradation (e.g., P-T profile, composition, predicted condensate, and wall loss vs. time)	Enable predictive maintenance Support risk management	Need highly detailed plant data and validated sub-models; Still emerging for CCS pipelines.
Online impurity and moisture monitoring	Real-time tracking of CO_2_ composition (H_2_O, SO_X_, NO_X_, O_2_, H_2_S, organics) at key nodes	Provides early warning of off-spec events; Supports implementation of quality controls and stream management	Sampling can cause in-line reactions and phase changes; Highly analytical cost and maintenance requirements.
Electrochemical and weight-loss corrosion monitoring (coupons, ER, LPR, EIS)	In situ corrosivity and corrosion rate at selected locations	Direct measurement of corrosion Capture transient spikes linked to acid dropout events; Established technology.	Localized representation only Interpretation complicated by multiphase flow and deposits; LPR and EIS require conductive aqueous phase.
In-line inspection (MFL, UT, caliper pigs)	Spatial distribution and depth of internal metal loss, pits, and geometry changes along pipelines	High-resolution mapping of accumulated damage; Support model validation and risk assessments	Periodic monitoring rather than continuous; Cannot distinguish acid dropout corrosion from other internal corrosion
Leak detection and fiber optic/external sensing	Detect loss of containment, pressure/flow anomalies, acoustic or temperature signatures of leaks.	Rapid leak detection and localization, including CO_2_-specific dispersion behavior	Detects damage after it has already occurred

## Data Availability

No new data were created or analyzed in this study. Data sharing is not applicable to this article.
